# *Rvi4* and *Rvi15* are the same apple scab resistance genes

**DOI:** 10.1007/s11032-023-01421-0

**Published:** 2023-10-11

**Authors:** Andreas Peil, Nicholas P Howard, Simone Bühlmann-Schütz, Ines Hiller, Henk Schouten, Henryk Flachowsky, Andrea Patocchi

**Affiliations:** 1 Julius Kühn Institut (JKI)—Federal Research Centre for Cultivated Plants, Institute for Breeding Research on Fruit Crops, Pillnitzer Platz 3a, 01326 Dresden, Pillnitz Germany; 2 Fresh Forward Breeding and Marketing B.V., Hogewoerd 1C, 6851 ET Huissen, The Netherlands; 3 Research Division Plant Breeding, Agroscope, Müller-Thurgau-Strasse 29, 8820 Wädenswil, Switzerland; 4 Department of Plant Breeding, Wageningen University & Research, Droevendaalsesteeg 1, P.O. Box 386, 6700 AJ Wageningen, The Netherlands

**Keywords:** *Malus* x *domestica*, *Venturia inaequalis*, Resistance breeding, Marker-assisted selection

## Abstract

The apple (*Malus* x *domestica*) scab (*Venturia inaequalis*) resistance genes *Rvi4* and *Rvi15* were mapped to a similar region on the top of linkage group 2 and both resistance genes elicit the same type of resistance reaction, i.e., a hypersensitive response; hence, it is suspected that the two genes may be the same. As the two resistance genes *Rvi4* and *Rvi15* are currently used in apple breeding, it is important to clarify whether the two resistance genes are the same or not. Several approaches were used to make this determination. First, the pedigree of the genotype GMAL 2473, the source of *Rvi15*, was reconstructed. GMAL 2473 was found to be an F1 of ‘Russian seedling’, the genotype, which is known to also be the source of *Rvi4*. Next, it was further demonstrated that ‘Regia’, a cultivar known to carry *Rvi4* (and *Rvi2*), carries the same gene (*Vr2-C*), which was demonstrated to be the gene inducing *Rvi15* resistance. Finally, it was shown that transgenic lines carrying *Vr2-C* are compatible with race *4* apple scab isolates. Taken all together, these results definitively demonstrate that *Rvi4* and *Rvi15* are the same resistance gene. For future studies, we suggest referring to this resistance with the first name that was assigned to this gene, namely *Rvi4*.

**Supplementary Information:**

The online version contains supplementary material available at 10.1007/s11032-023-01421-0.

## Introduction

Apple scab, caused by the ascomycete *Venturia inaequalis* (Cooke) G. Winter, is the most important fungal disease in apple production in regions with frequent rain during spring (Belete and Boyraz 2017). The control of the disease in conventional, integrated, and biological production is based on the frequent application of fungicides (Zaller et al. 2023). The use of scab resistant cultivars has the potential of reducing the dependence on these products. The large majority of apple scab resistant cultivars that are currently available carry the apple scab resistance gene *Rvi6* (formerly *Vf*) originating from the wild apple *Malus floribunda* 821 (Khajuria et al. 2018). Unfortunately, this resistance has been overcome by the pathogen in Europe (Parisi et al. 2003, Parisi et al. 2004; Patocchi et al. 2020) and in the USA (Beckerman et al. 2009; Papp et al. 2020). The breakdown of *Rvi6* intensified the efforts of breeders to use other resistance genes and to combine them to produce cultivars with more durable resistance.

Only a limited number of commercial cultivars carrying apple scab resistance genes other than *Rvi6* are available and few cultivars carrying scab resistance gene pyramids have been developed so far. Examples are ‘Reka’, which carries *Rvi2*; ‘Regia’, ‘Realka’, ‘Rea Agatav’, and ‘Rea Bellina’, which carry the combination of *Rvi2* and *Rvi4*; and ‘Rea Cadis’, which carries a combination of *Rvi4* and *Rvi6*. To date, numerous scab resistance genes have been identified, 17 of which have been included in the *Rvi* nomenclature system (Bus et al. 2011). A summary of the first 10 years of the VINQUEST initiative (Patocchi et al. 2020) reported that most of these single genes were overcome. Exceptions were *Rvi11* (formerly *Vbj*) from the wild apple genotype *Malus baccata* jackii (Gygax et al. 2004) and *Rvi15* from GMAL 2473 (alias *Vr*_*2*_, Patocchi et al. 2004) that were reported as not overcome. *Rvi15* was considered to be the most promising resistance gene that could be incorporated relatively quickly into a new cultivar in combination with other scab resistance gene(s) for durable resistance because the fruits of its source GMAL 2473 are of much better quality (e.g., fruit size of about 6 cm measured in August, Patocchi A. unpublished) than those of *Malus baccata* jackii (fruit size of ca. 1.5 cm measured in August).

*Rvi15* is a resistance gene that induces pin-point pits (Galli et al. 2010a). This gene has been mapped on the top of linkage group (LG) 2 (Patocchi et al. 2004) and then identified via positional cloning. Galli et al. (2010b and c) found three putative resistance genes (*Vr2-A*, *Vr2-B*, and *Vr2-C*) characterized by a toll and mammalian interleukin-1 receptor protein nucleotide-binding site leucine-rich repeat structure (TIR_NBS_LRR) on the BAC clone spanning the resistance region of *Rvi15*. The three genes *Vr2-A*, *Vr2-B*, and *Vr2-C* had coding sequences of 3273 bp (1091 amino acids), 4032 bp (1344 amino acids), and 3296 bp (1099 amino acids), respectively, and showed differences in all five domains, the most evident being in the length of the leucine-reach repeat domain (Galli et al. 2010c). In a subsequent study, Schouten et al. (2014) functionally demonstrated that the candidate gene *Vr2-C* was the gene promoting *Rvi15* resistance. Recently, several *Rvi15* ‘Gala Galaxy’ trans- and cisgenic lines have been developed (Giovanni Broggini personal communication).

*Rvi4*, named *Vh4* until the nomenclature of the apple scab resistance genes was revisited in Bus et al. (2011), is a resistance gene that also induces pin-point pits (Bus et al. 2005). Interestingly, Bus et al. (2005) mapped *Rvi4*, which is derived from R12740-7A (alias ‘Russian seedling’, Dayton et al. 1953), to the top end of the LG 2, very closely to *Rvi15*, but at a different map position. In fact, in Galli et al. (2010b), *Rvi15* mapped to a position 2 cM above the SSR marker CH02c02c, while *Rvi4* was mapped to 5 cM below the same marker in Bus et al. (2005). Yet, the mapping of *Rvi4* conducted by Bus et al. (2005) was refined in the work of Jänsch et al. (2015). Checking the original molecular data of Bus et al. (2005), genotyping mistakes were found, and their correction led to *Rvi4* being remapped to 0.4 cM above CH02c02c. With this new information, amplicons originally developed for *Rvi15* were searched for SNPs associated with *Rvi4*. Fifty-three SNPs, all co-segregating with *Rvi4*, were found (Jänsch et al. 2015).

With *Rvi4* and *Rvi15* now mapping to the same region, both inducing a hypersensitivity response (HR), and both being of particular interest for the breeders, it was necessary to definitively prove whether or not the two apple scab resistance genes are indeed the same.

To answer this question, (1) the alleles of the molecular markers linked with/in physical proximity of *Rvi4* and *Rvi15* amplified from *Rvi4* and *Rvi15* genotypes were compared, (2) part of the pedigree of GMAL 2473 was reconstructed and the haplotypes of cultivars/selections with *Rvi2*, *Rvi4*, both resistances, and ‘Russian Seedling’ were compared, (3) amplicons spanning the whole *Vr2-C* coding region of ‘Regia’ (*Rvi2* and *Rvi4*) were sequenced and compared with those of GMAL 2473 (*Rvi15*), and (4) *Rvi4* and *Rvi15* genotypes were inoculated with apple scab isolates known to overcome *Rvi4* resistance.

## Materials and methods

### Plant material and DNA extraction

GMAL 2473 used in this study was the same as used in Patocchi et al. (2004), Galli et al. (2010a), Galli et al. (2010b), Galli et al. (2010c), and Jänsch et al. (2015). It originated from the Geneva National Germplasm Repository (PI 589835). This genotype is not available anymore at the US repository. Selections 04E1-84, 04E1-860, 04E1-595, 04E1-918, and 04E1-33 are progeny of a ‘Golden Delicious’ x GMAL 2473 cross previously used by Galli et al. (2010b). These seedlings, the only still available of this cross, were used for phasing the SNP markers alleles of GMAL 2473.

The leaf tissue of ‘Regia’, TSR33T239, and GMAL 2473 that was used for the molecular marker analysis derived from the collection of genotypes of Agroscope Wädenswil (Switzerland). The leaf tissue of ‘Regia’ and GMAL 2473 that was used for the sequencing of *Vr2-C* genomic DNA and cDNA derived from trees of the collection of JKI Dresden-Pillnitz (Germany). The seven transgenic lines Vr 2c-603/1, Vr 2c-604/1, Vr 2c-608/1, Vr 2c-610/1, and Vr 2c-612/1 used for the scab inoculation test were those developed by Schouten et al. (2014). Control genotypes of this latter experiment were ‘Regia’, ‘Gala’, and ‘Gala’ wild type (wt) used for the generation of the transgenic lines, TSR33T239 and GMAL 2473 of the collection of JKI Dresden-Pillnitz (Germany). All the not genetically modified genotypes were grown in the field, while the transgenic lines were grown in a greenhouse under standard conditions.

Genomic DNA of field- or greenhouse-grown plants was extracted from about 100 mg of fresh leaf tissue using the DNeasy® Plant Mini Kit (Qiagen, Hilden, Germany) according to manufacturer’s instructions.

### Isolates for scab inoculation tests

Three different *Venturia inaequalis* isolates were used in this study: 104 (race 1), 1634 (race 4), and Regia2 (race 2, 4). Isolate 104 was isolated from ‘Golden Delicious’ (Guillaumès et al. 1995), whereas isolate 1634 was isolated from TSR33T239, a genotype carrying *Rvi4* (Caffier et al. 2015). Regia2 was isolated from ‘Regia’, which carries both resistance genes *Rvi2* and *Rvi4*. The latter isolate is therefore virulent to both *Rvi2* and *Rvi4* (Peil et al. 2018).

### Molecular marker analysis

The curated SNP array data and the sources for each accession used are listed in supplementary tables 1 to 3 (online resource 1). SNP data from the 20K SNP genotyping array (Bianco et al. 2014) was generated for some individuals in this study, made available from previous studies, or shared for use in this study for pedigree reconstruction and haplotype tracing. Data for 04E1-84, 04E1-860, 04E1-595, 04E1-918, and 04E1-33 was generated by Agroscope. SNP array data for ‘Regia’, ‘Reka’, and their common parent PRI 388-14 (Andreas Peil and Eric van de Weg unpublished, also known as OR40T43) was made available from the FruitBreedomics project (Laurens et al. 2018). SNP array data for the three offspring of ‘Russian Seedling’ were made available from the Palouse wild cider apple breeding program (James et al. 2022) and used for phasing the SNP data of ‘Russian Seedling’. The SNP array data was curated following the methods described in Vanderzande et al. (2019) using the set of 10,321 robust SNPs used in Volk et al. (2022).

Pedigree reconstruction for GMAL 2473 and pedigree validation for ‘Regia’, ‘Reka’, and their pedigree ancestors were conducted using the methods described in Vanderzande et al. (2019) and Howard et al. (2021). Parent-offspring duo relationships were ordered using the parent-offspring order resolution test (Howard et al. 2022). Candidate pedigree ancestors of GMAL 2473 were considered from a panel of over 5000 cultivars and germplasm accessions from an ongoing, large-scale collaborative pedigree reconstruction project (Howard et al. 2018a). SNP array data was phased using FlexQTL (Bink et al. 2014). Phased data for ‘Russian Seedling’, GMAL 2473, PRI 388-14, ‘Regia’, and ‘Reka’ were compared to demonstrate identity by descent over the regions of chromosome 2 containing *Rvi2* and *Rvi4*.

The physical positions for *Rvi2* and *Rvi4* were estimated by BLASTing sequences associated with them against the *Malus* x *domestica* HFTH1 Genome v1.0.a1 assembly (Zhang et al. 2019). For *Rvi2*, the sequences (KM104993 and KM105136, respectively) containing the SNP markers FBsnRvi2-5 and FBsnRvi2-6.1, which flank *Rvi2* (Jänsch et al. 2015), were used. For *Rvi4*/*Rvi15*, the primer sequence Vr2Cfull_fw was used as the position of this sequence on HFTH1 was found to be close to those of the marker FBsnRvi4.1 co-segregating with *Rvi4* in Jänsch et al. (2015).

The physical coordinates retrieved were compared to the 10,321 robust SNPs on the SNP array to identify flanking SNPs to use as a focal point for building haplotypes covering *Rvi2* and *Rvi4* to use for haplotype tracing as described in Howard et al. (2018b).

The *Rvi4* and *Rvi15* molecular markers (SSR and KASP™) listed in supplementary tables 4 and 5 (online resource 2) were tested on DNA of the genotypes GMAL 2473 (*Rvi15*), ‘Regia’ (*Rvi2* and *Rvi4*) and ‘Gala’. ‘Gala’ was included to support the transfer of the SSR markers in other laboratories (correction of possible shift of the allele sizes).

SSR analyses were conducted at Ecogenics GmbH (Balgach, Switzerland), while SNP genotyping using the competitive allele specific PCR genotyping system (KASP™) was conducted at LGC Genomics Ltd. (Teddington, UK).

### Sequencing of *Vr2*-C genomic DNA and cDNA from *Rvi4* and *Rvi15* genotypes

The primers Vr2C_full_fw and Vr2C_full_II_rev were used for full-length amplification of *Vr2-C* from ‘Regia’ and GMAL 2473. The PCR was performed in 1× Long Range PCR buffer containing 10 ng template DNA, 5 μl dNTP’s (0.2 mM of each), 2.5 μl of each primer (10 μm), 0.25 μl Long Range PCR Enzyme Mix (Thermo Fisher Scientific, Erlangen, Germany), and ddH_2_O in a total volume of 50 μl. After denaturation for 3 min at 94 °C, 10 PCR cycles were performed with 20 s at 94 °C, 30 s at 70 °C, and 6 min at 68 °C. Subsequently, 25 PCR cycles were performed with 20 s at 94 °C, 30 s at 70 °C, and 6 min at 68 °C with a time increment of 5 s per cycle. After final extension at 68 °C for 10 min, the reaction was stored at 10 °C.

*Vr2-C* cDNA from ‘Regia’ and GMAL 2473 was synthetized as follows: young leaf tissue was homogenized using the Retsch Mixer Mill MM400 (Retsch, Haan, Germany). The total RNA was extracted using the InviTrap Spin Plant RNA Mini Kit (Stratec Molecular GmbH, Berlin, Germany). Genomic DNA contaminations were removed with the DNA-Free Kit (Life Technologies GmbH, Darmstadt, Germany). For cDNA synthesis, the RevertAid™ First Strand cDNA Synthesis Kit (Thermo Scientific, Braunschweig, Germany) was used and 1 μg of total RNA as template. RT-PCR for detecting full-length *Vr2C* transcripts was performed using the primers Vr2C_full_fw and Vr2C_full_II_rev.

Amplified RT-PCR products were separated on a 0.8–1.0% agarose gels, isolated and ligated into the vector PCRTM2.1-TOPO^TM^ (Invitrogen, Groningen, The Netherlands), and transferred into One Shot^TM^ cells according to the manual. Plasmids for sequencing were isolated with the QIAGEN Plasmid Mini Kit (QIAGEN, Hilden, Germany). Sequencing was performed at Eurofins Genomics (Ebersberg, Germany) using the sequencing primers listed in Table 1.

**Table 1:** Primers used to amplify and sequence *Vr2-C* (genomic and cDNA) from different *Rvi4* and *Rvi15* genotypes

Gene	Primer name	F/R	Sequence 5′→3′
Vr2C	Vr2C5′UTR_F	F	CTTCCACTGAAATCAACACC
	Vr2C5′UTR_R	R	AGGAAAGAGAGAGGCAAAAA
	Vr2Cfull_fw	F	CTCTCTTTCCTTGCACCTTCCTGCCCGGTG
	Vr2Cfull_IIrev	R	TCAGATGCCAAAACTGATTCTACCTCTAGTGTCGCCC
	Vr2Cseq1	F	GATGTATGCGGATTCCAGTTGG
	Vr2Cseq2	F	GGATTGGGTAAAACAACAGTTGCC
	Vr2Cseq3	F	GGGACAAGGACTACGTTGCAAA
	Vr2Cseq4	F	ATGGTTGCGTTGGGAAGGATG
	Vr2Cseq5	F	CTCACATACGGCGCATTAGA
	Vr2Cseq6 (M13F_uni43)	F	AGGGTTTTCCCAGTCACGACGTT
	Vr2Cseq7 (M13R_rev49)	R	GAGCGGATAACAATTTCACACAGG
	Vr2Cseq8	F	CTCCTCTTCGTCCTCCTCT
	Vr2Cseq9	F	CCTTGTCCACATCATCATT
	Vr2Cseq10	F	ACCACCAGACAACTGACAAC
	Vr2Cseq11	F	GGTAAAATGGTTGTGGTTG
	Vr2Cseq12	F	GCGTCAATTCCTCAGCTTC

### Scab inoculation

Conidia for inoculation were propagated as described by Peil et al. (2018) with small modifications for isolates 104 and 1634. Isolates 104 and 1634 were additionally propagated on plates containing 0.75% malt extract. Conidia were washed from the cellophane membranes and diluted with water to a concentration of 1.5–2.5 × 10^5^ conidia/ml. Up to five replicates per genotype were produced by grafting budwood on the M9 rootstock (online resource 4). Plants were grown in a ventilated greenhouse (18°C day/15 °C night) for about 9 weeks. The youngest leaves of actively growing shoots were spray inoculated as described in Peil et al. (2018). Three replicates of each genotype were inoculated with isolate 104. Five replicates of each transgenic line and GMAL2473 and one replicate of ‘Regia’ were inoculated with isolate 1634. Five replicates of each transgenic line and ‘Regia’ and three replicates of GMAL2473 were inoculated with Regia2. Symptoms were scored after 21 and 28 day post-inoculation (dpi) according to (Chevalier et al. 1991). Score values were as follows: 0—no symptoms, 1—pin point pits, 2—chlorosis, 3a—necrotic and some chlorotic lesions with very slight occasional sporulation, 3b—clearly sporulating chlorotic and necrotic lesions, and 4—sporulating lesions.

## Results

### Molecular marker analysis

SSR and SNP molecular markers previously reported to be associated with the resistance genes *Rvi4* and *Rvi15* have been tested on ‘Regia’ and GMAL 2473. The results for ‘Regia’ and GMAL 2473 were identical. In fact, both genotypes amplified with all markers the allele in coupling with the *Rvi4* or *Rvi15* resistance (Table 2).

**Table 2:** Results of the test of ‘Regia’ and GMAL 2473 with molecular markers associated to *Rvi4* and *Rvi15*. The molecular markers have been ordered according their position on the HFTH1 Genome v1.0.a1 genome (Zhang et al. 2019)

Marker	Marker type	Position of the start of the sequence containing the SNP or the SSR repeat^1^	Allele in coupling with *Rvi4/15*	Alleles of ‘Regia’ (*Rvi2 and 4*)	Alleles of GMAL 2473 (*Rvi15*)	Alleles of ‘Gala’
Hi22d06	SSR	1315387	132	**132**	**132**	**132**/138
CH02f06	SSR	1936045	155	**155**/159	**155**/157	147/167
FBsnRvi4.1_K146^2^	SNP	2844103	T	**T**G	**TT**	GG
Est. position *Rvi4/15*^3^		2874817				
21k14t7_Rvi15_R153	SNP	2973729	G	**GG**	**G**A	AA
CHsnRvi15.1_S188	SNP	3188014	G	**GG**	**G**C	CC
CH02c02a	SSR	3467978	182	163/**182**	**182**/186	148/184

^1^SSR markers sequences were retrieved from the HiDRAS website (unimi.it), while SNP marker sequences were published by Jänsch et al. (2015). These sequences were blasted against the *Malus* x *domestica* HFTH1 Genome v1.0.a1 chromosomes (Zhang et al. 2019) at the Genome Database for Rosaceae (GDR, https://www.rosaceae.org/). The position on the HFTH1 Genome v1.0.a1 of the first base of the basted sequence is indicated and used to order the markers.

^2^According Jänsch et al. (2015) this marker co-segregates with *Rvi4*.

^3^The position of *Rvi4/Rvi15* has been estimated blasting the sequence of the primer Vr2Cfull_fw against the *Malus* x *domestica* HFTH1 Genome v1.0.a1 chromosomes (Zhang et al. 2019)

Bold: allele in coupling with the resistance gene *Rvi4* or *Rvi15*

### Pedigree reconstruction of GMAL 2473

GMAL 2473 and ‘Russian Seedling’ shared at least one allele at every locus, indicating that they have a parent-offspring relationship. ‘Russian Seedling’ was confirmed as the parent of GMAL 2473 via the parent-offspring order resolution test using the five offspring of GMAL 2473 (04E1_33, 04E1_595, 04E1_84, 04E1_860, and 04E1_918) that were included in this study. Roughly 30% of the haplotypes from the unknown parent of GMAL 2473 are identical to those in ‘Duchess of Oldenburg’, thus indicating ‘Duchess of Oldenburg’ as a likely grandparent of the unknown parent of GMAL 2473. This result provided further confirmation that GMAL 2473 was an offspring rather than the parent of ‘Russian Seedling’ because there were not haplotypes of ‘Duchess of Oldenburg’ in ‘Russian Seedling’, which would have been expected if the opposite relationship were true.

**Fig. 1:**
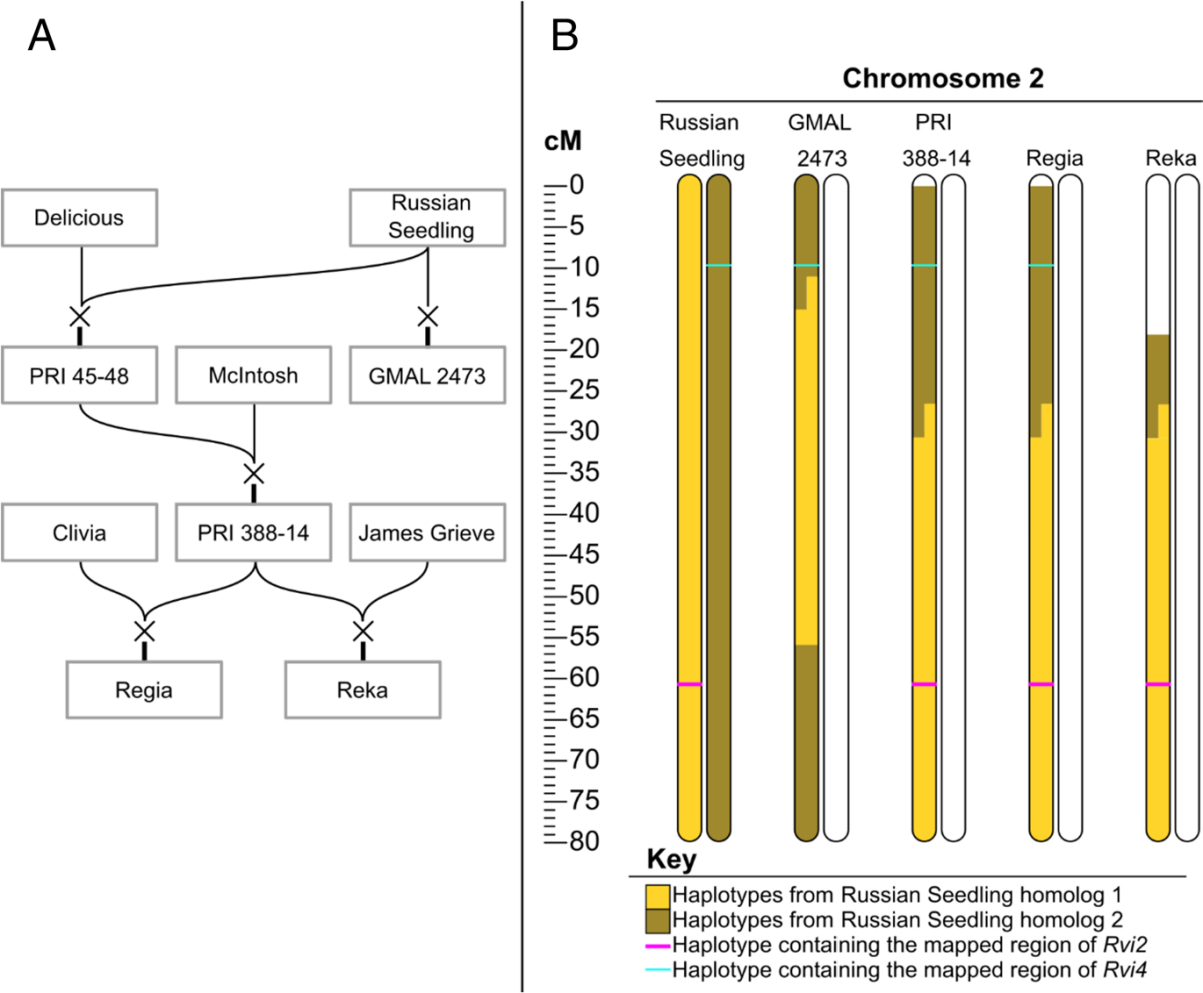
**A** Pedigree of descendants of ‘Russian Seedling’ and **B** depiction of chromosome 2 haplotypes of selected descendants of ‘Russian Seedling’, with the estimated locations of haplotypes containing the scab resistance genes *Rvi2* (pink) and *Rvi4* (green) highlighted

GMAL 2473 inherited the haplotype and the corresponding region harboring *Rvi4/15* from ‘Russian Seedling’ but did not inherit *Rvi2* due to a close recombination (Fig. 1). PRI 388-14 inherited both haplotypes containing the regions of *Rvi2* and *Rvi4* through its ungenotyped parent PRI 45-48, which matches its recorded pedigree of ‘Delicious’ x ‘Russian Seedling’. ‘Regia’ and ‘Reka’ inherited the haplotypes containing the region of *Rvi2* from PRI 388-14, though only ‘Regia’ inherited the region containing *Rvi4*.

### Sequencing of *Vr2-C* genomic DNA and cDNA of ‘Regia’ 

Full-length genomic DNA sequences of *Vr2-C* were amplified (amplicon of ~5.500 bp) from genomic DNA of GMAL 2473 and ‘Regia’ (amplicon of ~5.500 bp). The PCR product of ‘Regia’ was cloned and sequenced. The 5.594 bp long sequence (GeneBank: OR220343) was aligned to the sequence of the BAC clone 32A4 of GMAL 2473 (GenBank: KF055410.1). Both sequences were nearly identical. Differences were found at three different positions. Two nucleotides (AT) in a microsatellite repeat were missing (10 vs 11 AT repeats) in the sequence obtained from ‘Regia’ at position 2322–2323. Two nucleotides were missing in a region with a repetition of T (10 vs 12) at position 3706–3707, and one T was missing in a region with a repetition of T (7 vs 8) at position 4143 (Suppl. Fig. 1).

The amplicons of the full length coding sequence (CDS) of *Vr2-C* obtained from ‘Regia’ and GMAL 2473 had also the same size (data not shown). The PCR products were cloned, and eight clones of ‘Regia’ and six clones of GMAL 2473 were sequenced. Six clones of ‘Regia’ and four clones of GMAL 2473 were nearly identical. Two clones per genotype were slightly different. These clones appeared to be other splice variants. The sequences of the six ‘Regia’ clones and the four GMAL 2473 clones were aligned separately and consensus sequences for both genotypes were generated. The consensus CDS sequences (GeneBank: OR220341 and OR220342) were both 3.366 bp long and 100% identical. Subsequently, these CDS sequences were aligned to the GMAL 2473 BAC 32A4 sequence (GenBank: KF055410.1), the genomic *Vr2-C* sequence of ‘Regia’ and the predicted *Vr2-C* CDS sequence published by Galli et al. (2010a). Both CDS sequences (‘Regia’ and GMAL 2473) matched perfectly to the BAC 32A4 sequence. However, some differences were found to the predicted *Vr2-C* CDS of Galli et al. (2010b). The predicted *Vr2-C* CDS has a length of 3.288 bp and consists of eight exons and seven introns. The CDS sequences of ‘Regia’ and GMAL 2473 obtained in this study have a length of 3.366 bp and consist of six exons and five introns (Suppl. Fig. 1).

### Scab inoculation results

**Fig. 2:**
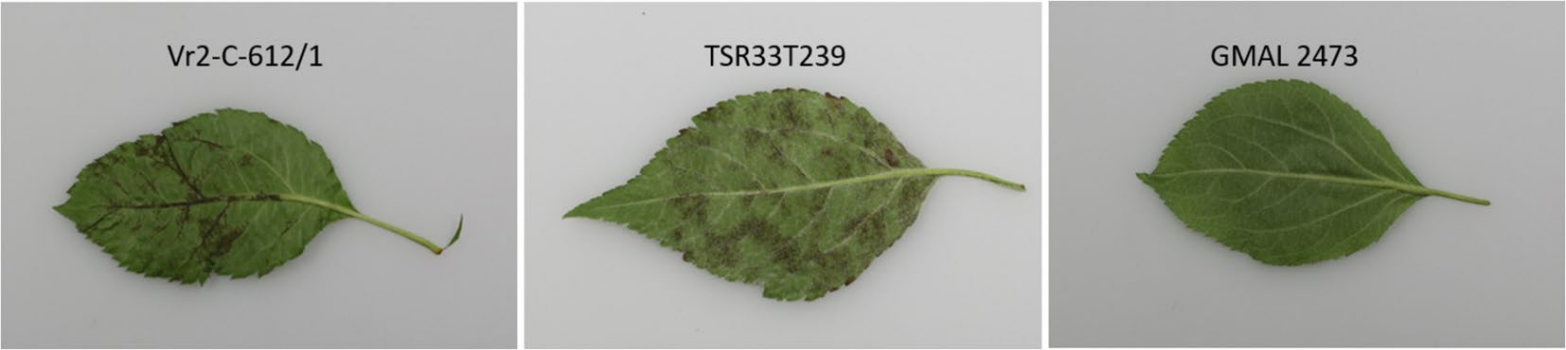
Inoculation results of the *Vr2-C* transgenic line 612/1, TSR33T239, and GMAL 2473 with the scab isolate 1634 28 dpi. Vr2-C-612/1 and TSR33T239 show strong apple scab sporulation while GMAL 2473 did not show any symptoms

All three tested scab isolates proved to be pathogenic as they infected ‘Regia’ and/or the apple genotype they were isolated from (Table 3, Suppl. Table 6, online resource 4). Isolate 104 (race 1), originating from ‘Golden delicious’ showed an incompatible reaction and induced a HR in GMAL 2473 and in all the *Vr2-C* transgenic lines. A compatible interaction (i.e., strong sporulation) was observed between isolate 1634 (race 4) and TSR33T239, as well as with all the *Vr2-C* transgenic lines (Fig. 2). Only ‘Regia’ and GMAL 2473 were incompatible with isolate 1634. Isolate Regia2 (race 2, 4) was compatible with all the tested genotypes with the exception of GMAL 2473, which showed chlorotic lesions. ‘Regia’

**Table 3:** Summary of the artificial scab inoculation of genotypes previously reported to carry *Rvi4,* *Rvi15* or *Rvi2 *and *Rvi4*. “Gala” was used as susceptible control. The highest score observed in the replicates is indicated.

Genotype	*Rvi* gene(s)		Scab isolates	
		104	1634	Regia2
Isolates from		GoldenD^1^	TSR33T239	‘Regia’
Race		1	4	2, 4
Year of inoculation		2014	2016	2015
‘Regia’	*Rvi2 Rvi4*	n.a.^2^	2	4
‘Gala’		4	(+)^4^	4
TSR33T239	*Rvi4*	(-)^3^	4	4
GMAL 2473	*Rvi15*	1	0	2
Vr2-C transgenic lines				
Vr 2c-603/1 (tree no. 70)	*Rvi15*	1	4	4
Vr 2c-604/1 (tree no. 73)	*Rvi15*	1	4	4
Vr 2c-608/1 (tree no. 74)	*Rvi15*	1	4	4
Vr 2c-610/1 (tree no. 77)	*Rvi15*	1	4	4
Vr 2c-612/1 (tree no. 82)	*Rvi15*	1	4	4
Gala wt of transgenic lines		4	(+)^4^	4

^1^GoldenD: Golden Delicious

^2^n.a., combination not analyzed

^3^(-) not tested, according Caffier et al. 2015 incompatible/resistant reaction

^4^(-) not tested, according Caffier et al. 2015 compatible/susceptible reaction

## Discussion

All the results produced in this work indicate that *Rvi4* and *Rvi15* are the same gene, the definitive evidence being the CDS of *Vr2-C* found in ‘Regia’, the *Vr2-C* transgenic lines losing their resistance when inoculated with isolates overcoming *Rvi4* resistance, the identification of ‘Russian Seedling’ (source of *Rvi4*) in the pedigree of GMAL 2473, and the demonstrated inheritance of haplotypes containing *Rvi4* in both ‘Russian Seedling’ and GMAL 2473.

Since the first mapping of *Rvi4* and *Rvi15* to similar positions on the top of LG2, and because both genes induced the same resistance reaction type (a rapid hypersensitive response), it had been suspected that the two genes might be the same. This presumption found first confirmations when Jänsch et al. (2015) discovered that both genes mapped in the same region of LG2, and when performing marker-assisted selection (MAS) for both genes, it was discovered that *Rvi4* and *Rvi15* genotypes amplified the same alleles in coupling with the two resistance genes (Table 2). These findings motivated us to definitively clarify whether the two genes were actually the same.

The reconstruction of the pedigree of GMAL 2473 and those of ‘Regia’ and ‘Reka’ showed that both *Rvi4* and *Rvi15* originated from the same source, i.e., ‘Russian seedling’, and that *Rvi2* and *Rvi4* are each located on the two different homolog chromosomes 2 in ‘Russian Seedling’. PRI 45-48, a direct offspring of ‘Russian Seedling’ and grandparent of ‘Regia’, inherited a recombinant chromosome 2 from ‘Russian Seedling’, resulting in PRI 45-48 having both resistances, i.e., *Rvi2* and *4* (Fig. 1A). PRI 45-48 transmitted most of this homolog, including both resistances, to PRI 388-14, which it then transmitted in full to its offspring ‘Regia’. However, since the two genes are relatively far apart (about 40 cM, estimated from the work of Bus et al. (2005) and about 51 cM in the iGL map used in this study for haplotype tracing), it is not surprising that ‘Reka’ inherited a recombinant homolog from PRI 388-14 that only contained *Rvi2*.

While the comparison of the full length CDS of *Vr2-C* of ‘Regia’ and GMAL 2473 showed no differences between each other and the genomic sequence of the BAC 32A4, differences of few nucleotides in three regions were observed between this sequence and the CDS predicted by Galli et al. (2010c). Differences between computer-calculated and experimentally studied CDSs occasionally occur (Vogt et al. 2013), and it is known that sequencing of transcripts usually improves the prediction (Minoche et al. 2015). Sequence differences were also observed while comparing the sequence of *Vr2-C* obtained from the BAC clone 32A4 and the consensus sequence of the cloned amplicons of *Vr2-C* from ‘Regia’. The three observed differences were all found in sequences known to be prone to sequences mistakes, i.e., microsatellites and relative long sequences of the same nucleotide. Moreover, these differences were observed in intronic regions, and therefore, they would not affect the function of the resistance gene. On the other hand, if real, these differences may be the results of mutations starting to accumulate over the generations.

The results of the scab inoculations done with race 1 (104) and race 4 (1634, Regia2) isolates clearly showed that all the genotypes carrying *Rvi4* and *Rvi15* were resistant (incompatible) to the race 1 isolate and susceptible (compatible) to race 4 isolates, which definitively confirmed that *Rvi4* and *Rvi15* are the same gene. One exception is GMAL 2473, that is resistant also to the race 4 isolates. This latter result is in line with the observations made in the context of the monitoring of the apple scab resistance gene breakdown of the VINQUEST initiative (Patocchi et al. 2020, http://www.vinquest.ch). In fact, so far, no compatible interaction on GMAL 2473 was observed over all the observation test sites, while for *Rvi4* compatible interactions have been observed in six test sites (in general, only few scab lesions found, Patocchi et al. 2020). Also, a study testing the differential hosts of VINQUEST with a set of different isolates (Caffier et al. 2015) reported that the differential host h(15) (GMAL 2473) was incompatible with all the isolates tested, while the differential host h(4) (TSR33T239) was compatible to three isolates, including 1634. This difference could be due to a second (yet unknown) resistance locus possibly present in GMAL 2473. The presence of this second resistance locus, possibly inducing partial resistance (QTL), may explain why Patocchi et al. (2004) could not define whether the 337 progeny of a cross of “Idared” x GMAL 2473 segregate for one or two resistance genes. In contrast, Galli et al. (2010b), studying the segregation between susceptible and resistant progeny plants of a ‘Golden Delicious’ x GMAL 2473 cross, clearly found a 1:1 ratio. One hypothesis explaining these contrasting results could be the different inoculum used; those of the second study being able to overcome the second unknown resistance factor, while this was not the case for the inoculum used for the first study.

Concluding, this study demonstrated that *Rvi4* and *Rvi15* are the same apple scab resistance gene. The two names *Rvi4* and *Rvi15* now refer to same resistance gene, but a single name should be used in the future. We propose to use *Rvi4*, as this name was the first assigned to this resistance and because it is probably more well known in apple breeding than *Rvi15*.

### Supplementary information

ESM 1 Supplementary Table 1 Apple genotypic profiles names (Analysis_Name), representative accessions (Accession_name) and associated accession IDs (Accession_ID) genotyped on the Illumina apple Infinium® 20K SNP array with the indicated genotypic profiles, the source of the SNP data (Data_source), and the collection site of the accessions sampled that were used for haplotype analysis and phasing in this study. Online Resource 1. Supplementary Table 2. Curated unphased SNP array data for genotypic profiles listed in Suppl. Table 1. Columns with numbers are the SNP index numbers from the Illumina apple Infinium® 20K SNP array. Online Resource 1. Supplementary Table 3. Curated phased SNP array data for genotypic profiles listed in Suppl. Table 1. Columns with numbers are the SNP index numbers from the Illumina apple Infinium® 20K SNP array. The column "VAR" denotes whether the haplotype data comes from the first (hap1) or second (hap2) parent. Online Resource 1. (XLSX 1926 kb)

ESM 2 Supplementary Table 4. Primers of SSR markers used for MAS for the genes *Rvi4* and *Rvi15.* Online Resource 2. Supplementary Table 5. Primers of the KASP assays used for MAS for the genes *Rvi4* and *Rvi15.* Online Resource 2. (PDF 163 kb)

ESM 3 Supplementary Table 6. Results of the artificial scab inoculation of the single replicates of the genotypes previously reported to carry *Rvi4* or *Rvi15* or a combination of both genes. 'Gala' was used as susceptible control. Online Resource 4. (PDF 9635 kb)

ESM 4 Supplementary figure 1. Sequence alignment of genomic and CDS sequences of *Rvi15* of 'Regia' and GMAL 2473. Rvi15_CDS_GMAL and Rvi15_CDS_Regia, sequences amplified from cDNA of 'Regia' and GMAL 2473 in this study, Rvi15_CDS_GMAL_Galli, CDS of *Rvi15* predicted by Galli et al. (2010c), Rvi15_genomic_Regia, genomic *Rvi15* DNA sequence of 'Regia' amplified and sequenced in this study, Rvi15_genomic_BAC32A4, and genomic DNA sequence of *Rvi15* obtained from the sequence of BAC32A4 (Galli et al. 2010a). Online Resource 3. (XLSX 14 kb)

## Data Availability

All the data supporting the results have been deposited in GeneBank (OR220341, OR220342 and OR220343) or can be found in the Supplementary information.
