# Supplementary material for: Rvi4 and Rvi15 are the same apple scab resistance genes
Source: Mol Breed. 2023 Oct 11;43(10):74. doi: 10.1007/s11032-023-01421-0 (PMC10564682; doi:10.1007/s11032-023-01421-0)
Supplement: Supplementary file 2 — Supplementary Table 4. Primers of SSR markers used for MAS for the genes Rvi4 and Rvi15. Online Resource 2. Supplementary Table 5. Primers of the KASP assays used for MAS for the genes Rvi4 and Rvi15. Online Resource 2. (PDF 163 kb) [file 11032_2023_1421_MOESM2_ESM.pdf]

Title: ***Rvi4* and *Rvi15* are the same apple scab resistance genes**
Journal: Molecular Breeding
Authors: Peil Andreas, Howard Nicholas P., Bühlmann-Schütz Simone, Ines Hiller, Schouten Henk, Flachowsky Henryk, Patocchi Andrea
Corresponding author: Andrea Patocchi, Research Division Plant Breeding, Agroscope, Müller-Thurgau-Strasse 29, 8820 Wädenswil, Switzerland; andrea.patocchi@agroscope.admin.ch

Suppl. Table 4 Primers of SSR markers used for MAS for the genes *Rvi4* and *Rvi15*

Marker	Primer name	F/R	Sequence 5'→3'
Hi22d06[1]	Hi22d06_F	F	CCCGAGCTCTACCTCAAA
	Hi22d06_R	R	CATTATGTTTCCGGTTTTTGG
CH02f06[2]	CH02f06_F	F	CCCTCTTCAGACCTGCATATG
	CH02f06_R	R	ACTGTTTCCAAGCGATCAGG
CH02c02a[2]	CH02c02a _F	F	CTTCAAGTTCAGCATCAAGACAA
	CH02c02a _R	R	TAGGGCACACTTGCTGGTC

[1] Silfverberg-Dilworth et al. 2006

[2] Liebhard et al. 2002

Suppl. Table 5 Primers of the KASP assays used for MAS for the genes *Rvi4* and *Rvi15*

Marker	Primer_AlleleX	Primer_AlleleY	Primer_Common	AlleleX	AlleleY
FBsnRvi4.1_K146	AATGTTACACCTGCATCTAAACACTC	CAATGTTACACCTGCATCTAAACACTA	GGAATGTTGACTGGATTAAGTTTATGGATT	G	T
21k14t7_Rvi15_R153	GGTAGTGGTGCAATGTGCTCTCA	GTAGTGGTGCAATGTGCTCTCG	CATCAACCTCCGCAATGGCATCTAT	A	G
CHsnRvi15.1_S188	GAACAGATTCATAAAGACTGATCAACC	GAACAGATTCATAAAGACTGATCAACG	GTGCATGTGATAAGATTTAAGTTGTGAGAA	C	G
