# Supplementary figures and images for: Rvi4 and Rvi15 are the same apple scab resistance genes
Source: Mol Breed. 2023 Oct 11;43(10):74. doi: 10.1007/s11032-023-01421-0 (PMC10564682; doi:10.1007/s11032-023-01421-0)

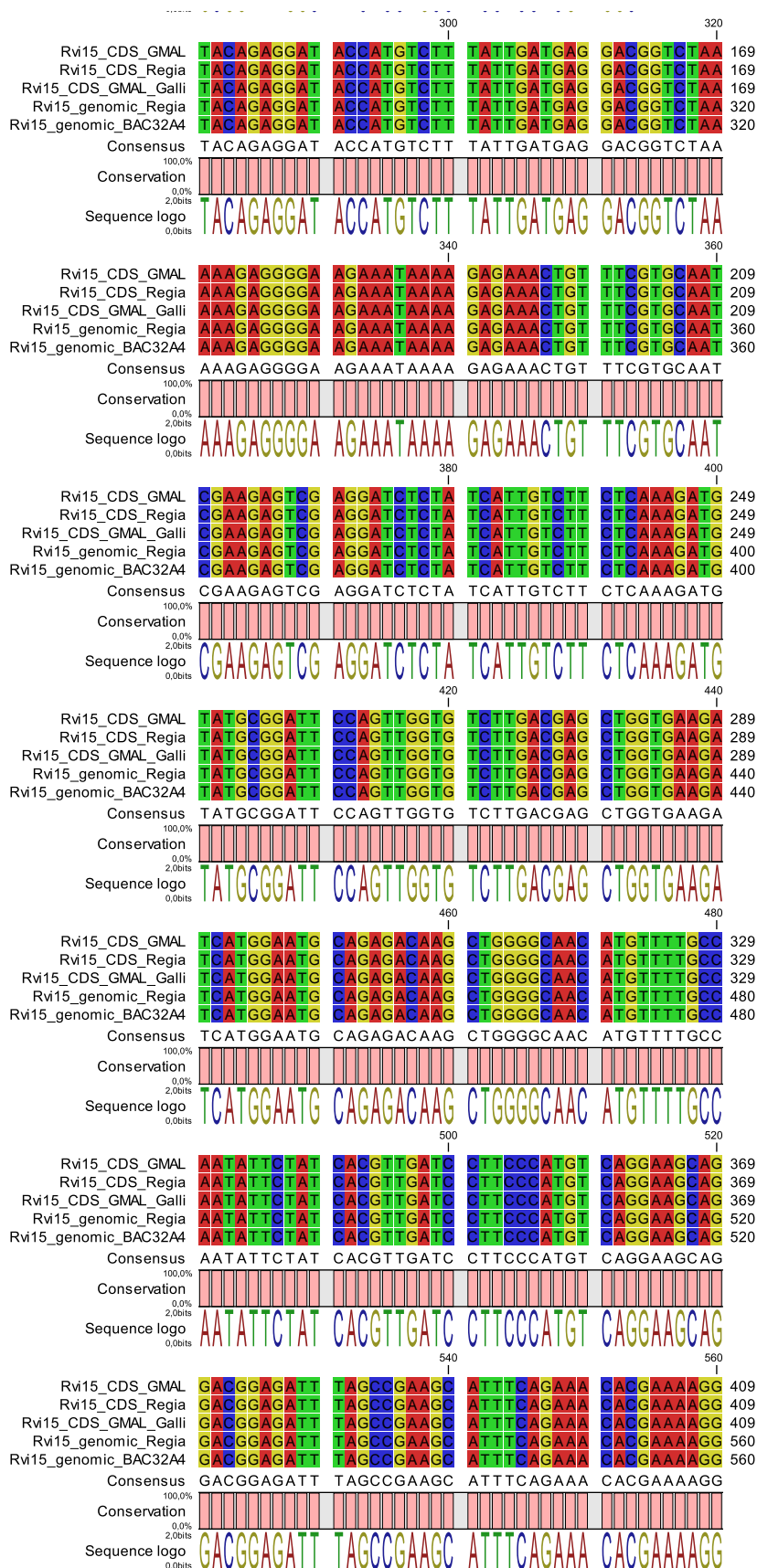

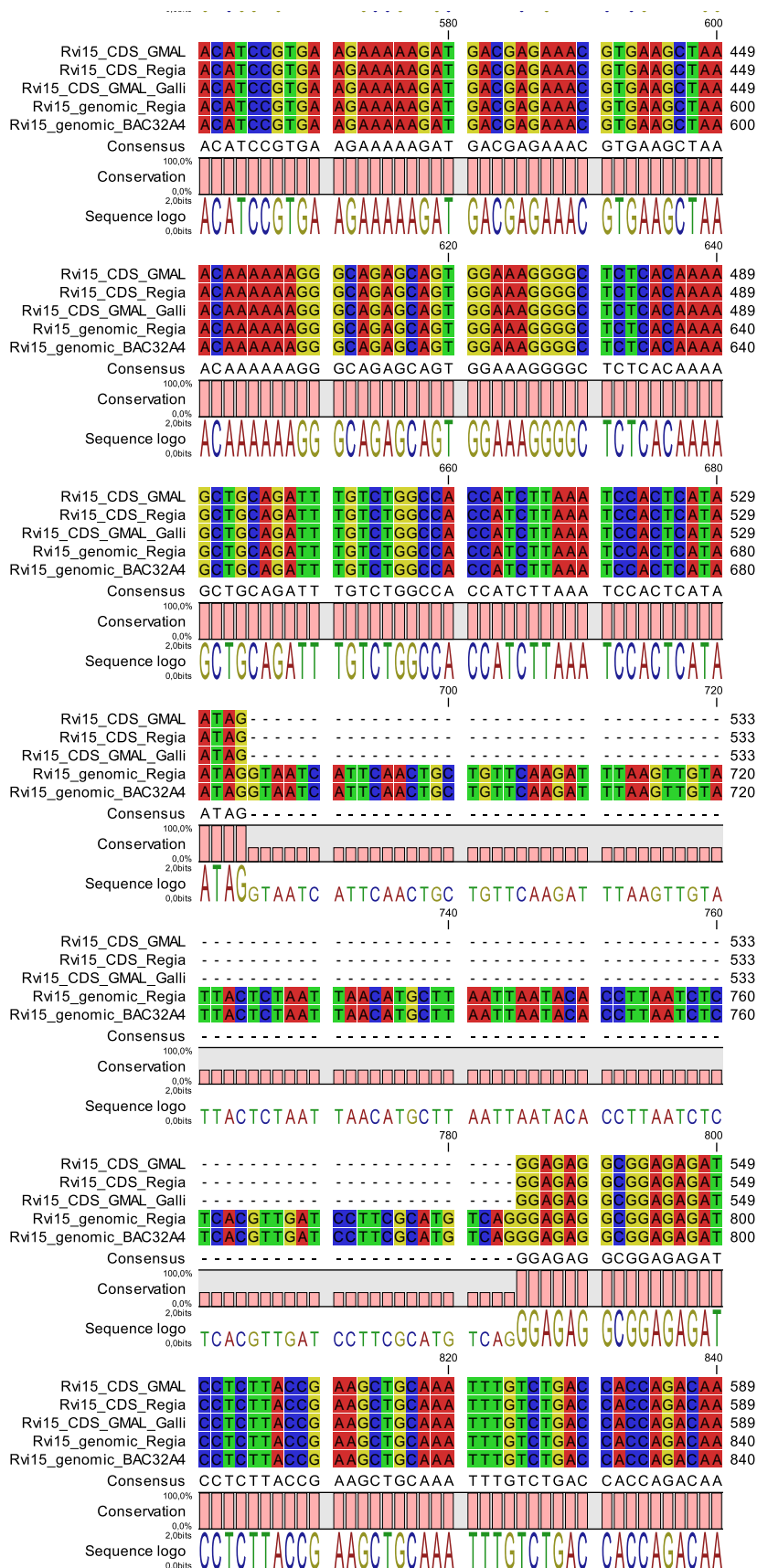

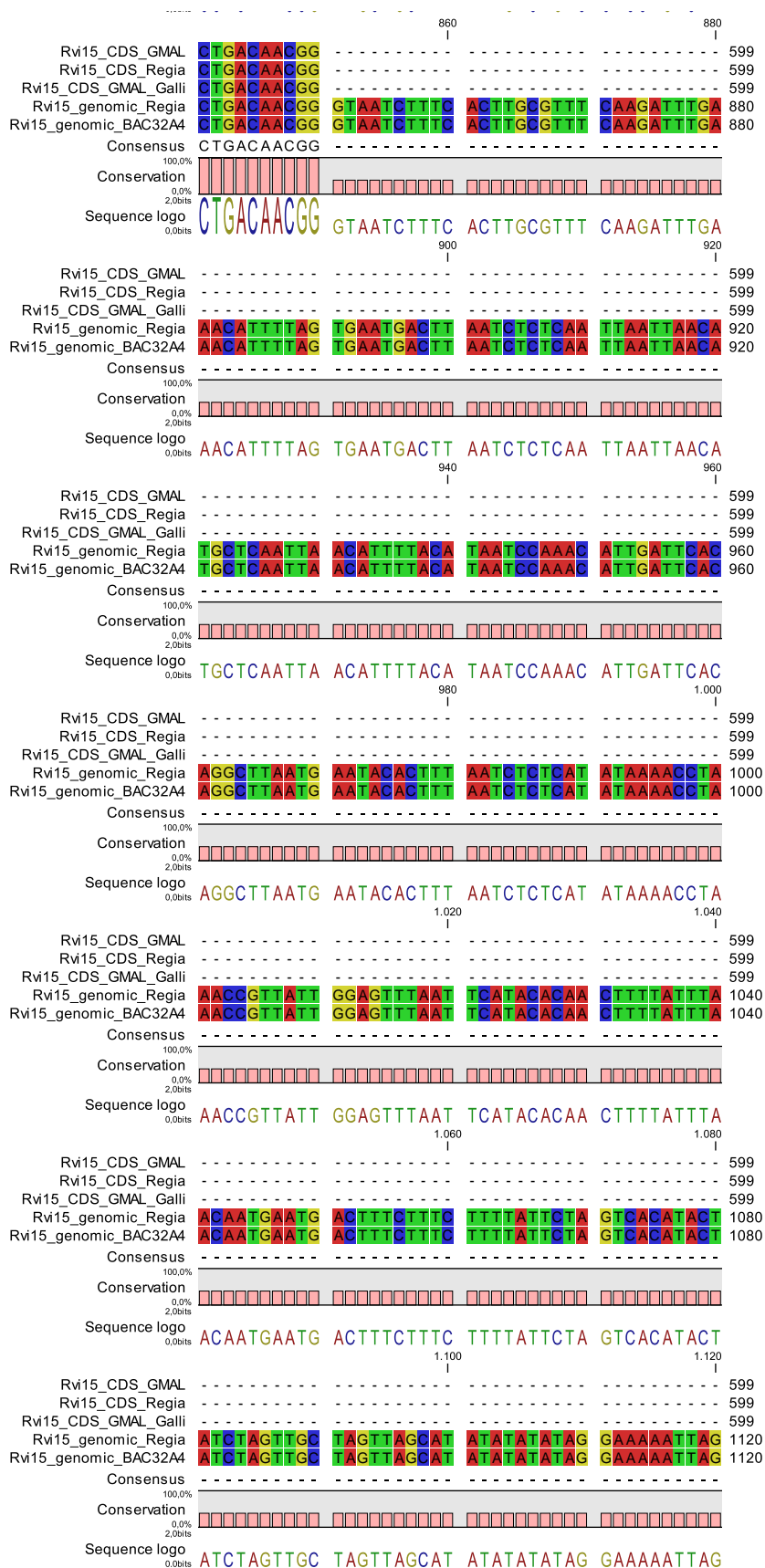

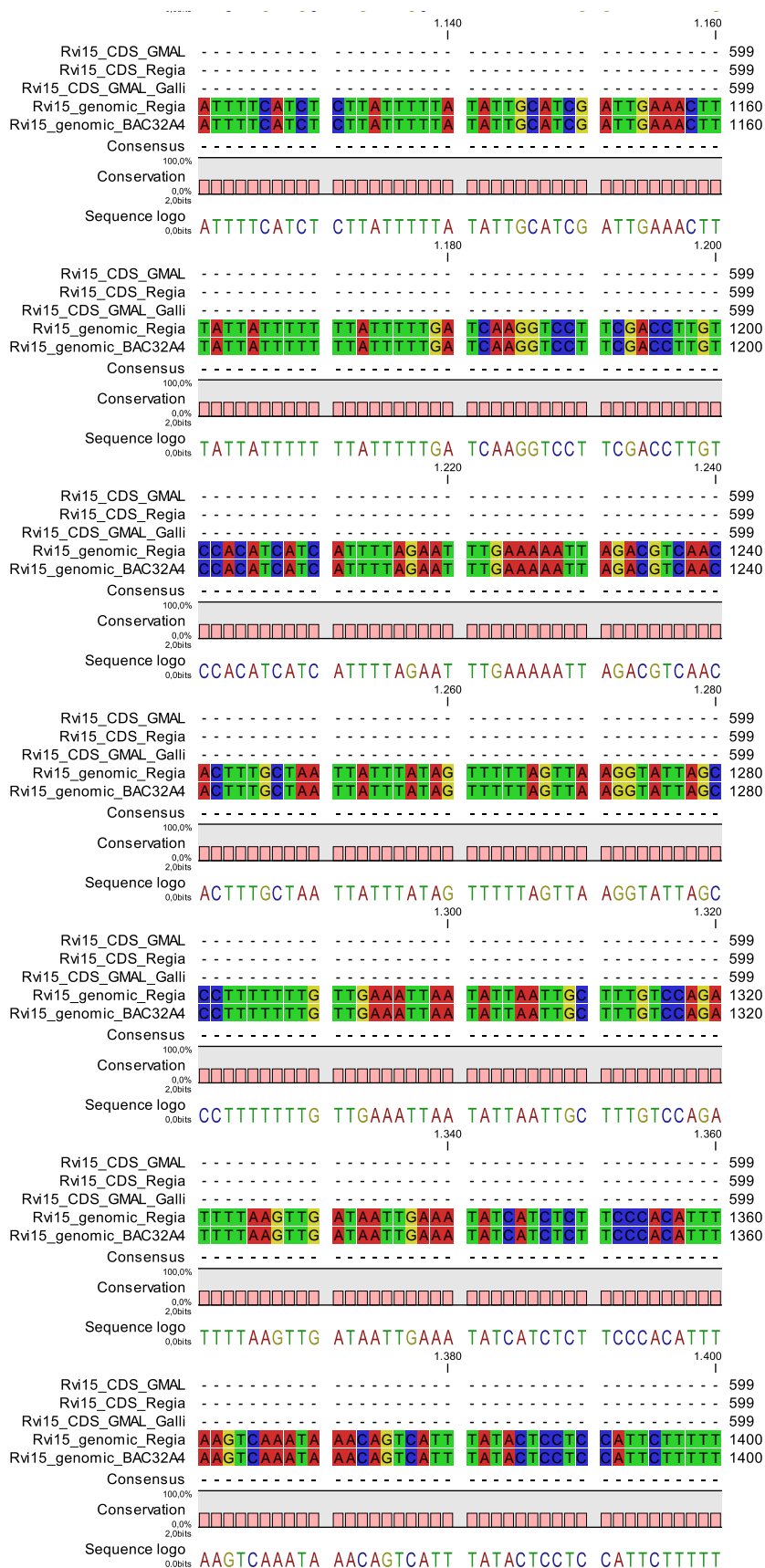

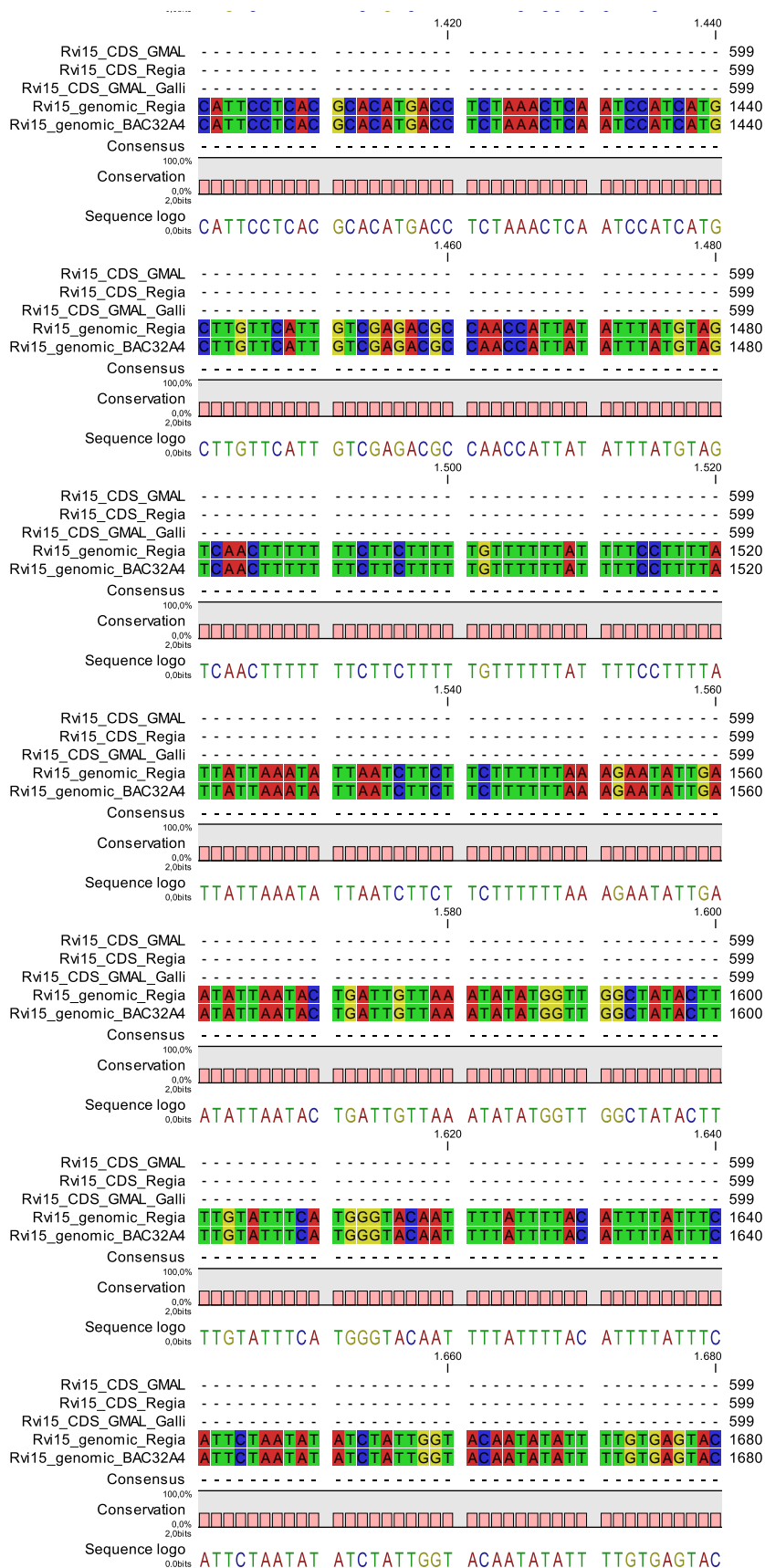

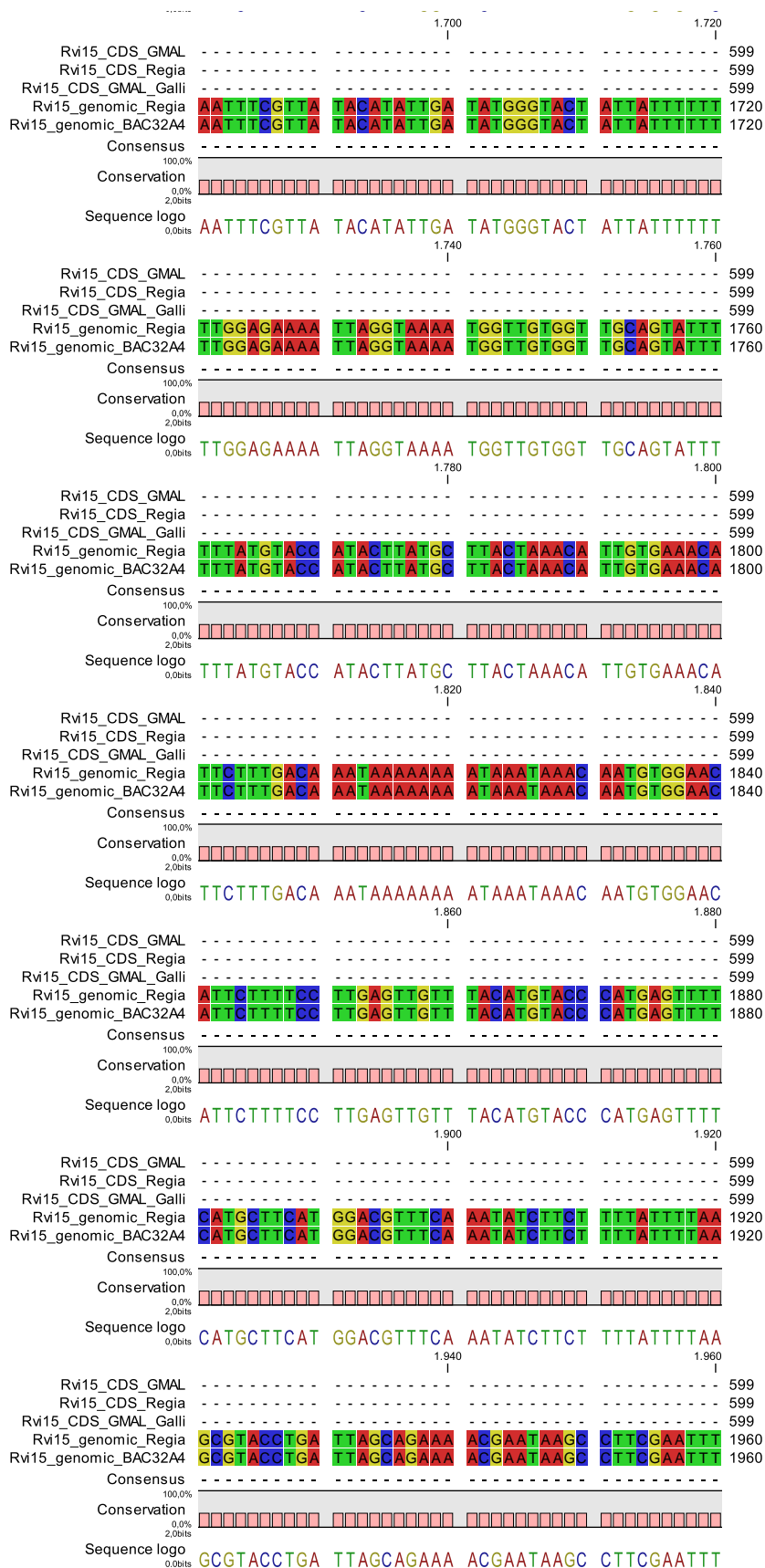

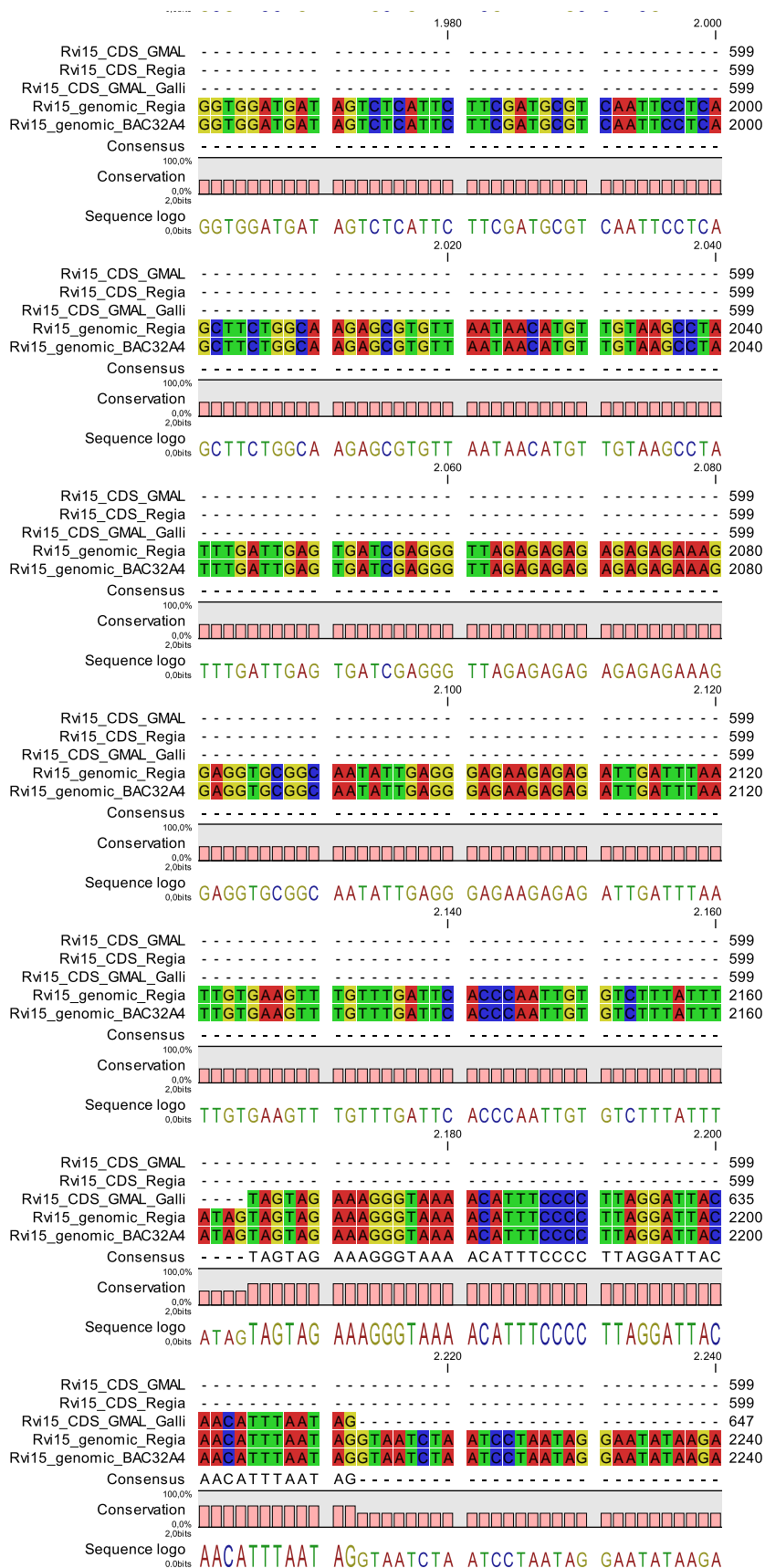

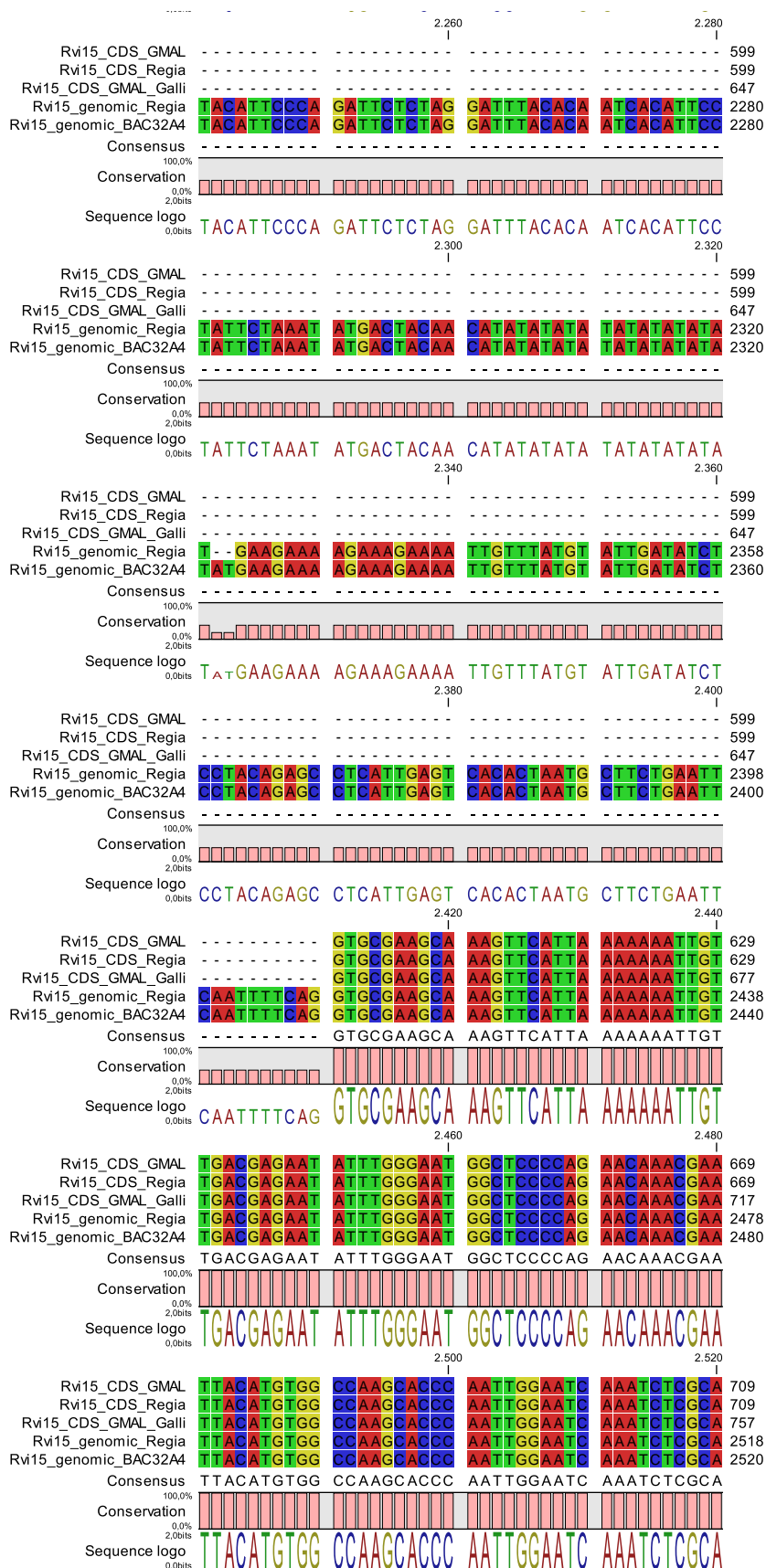

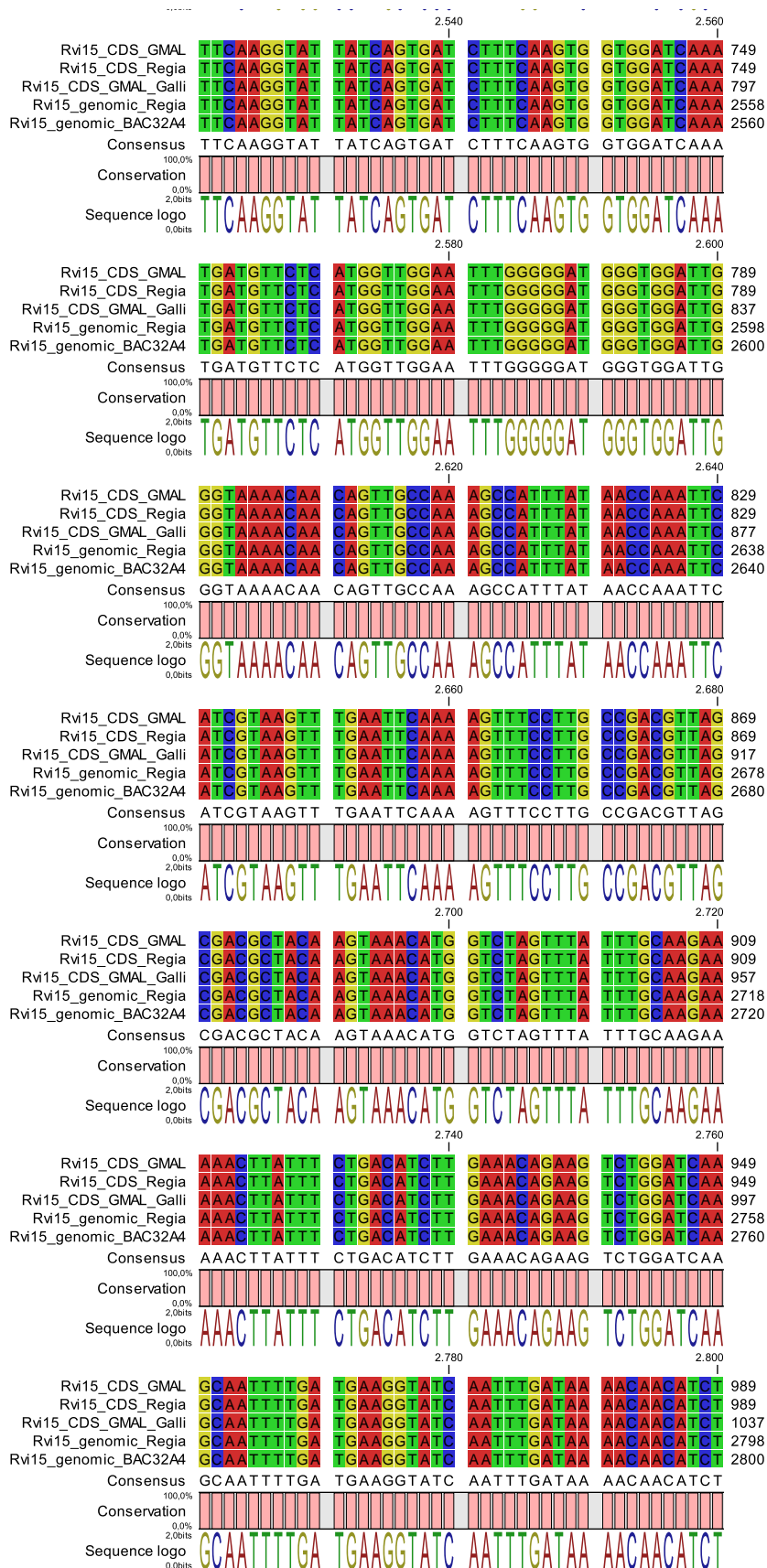

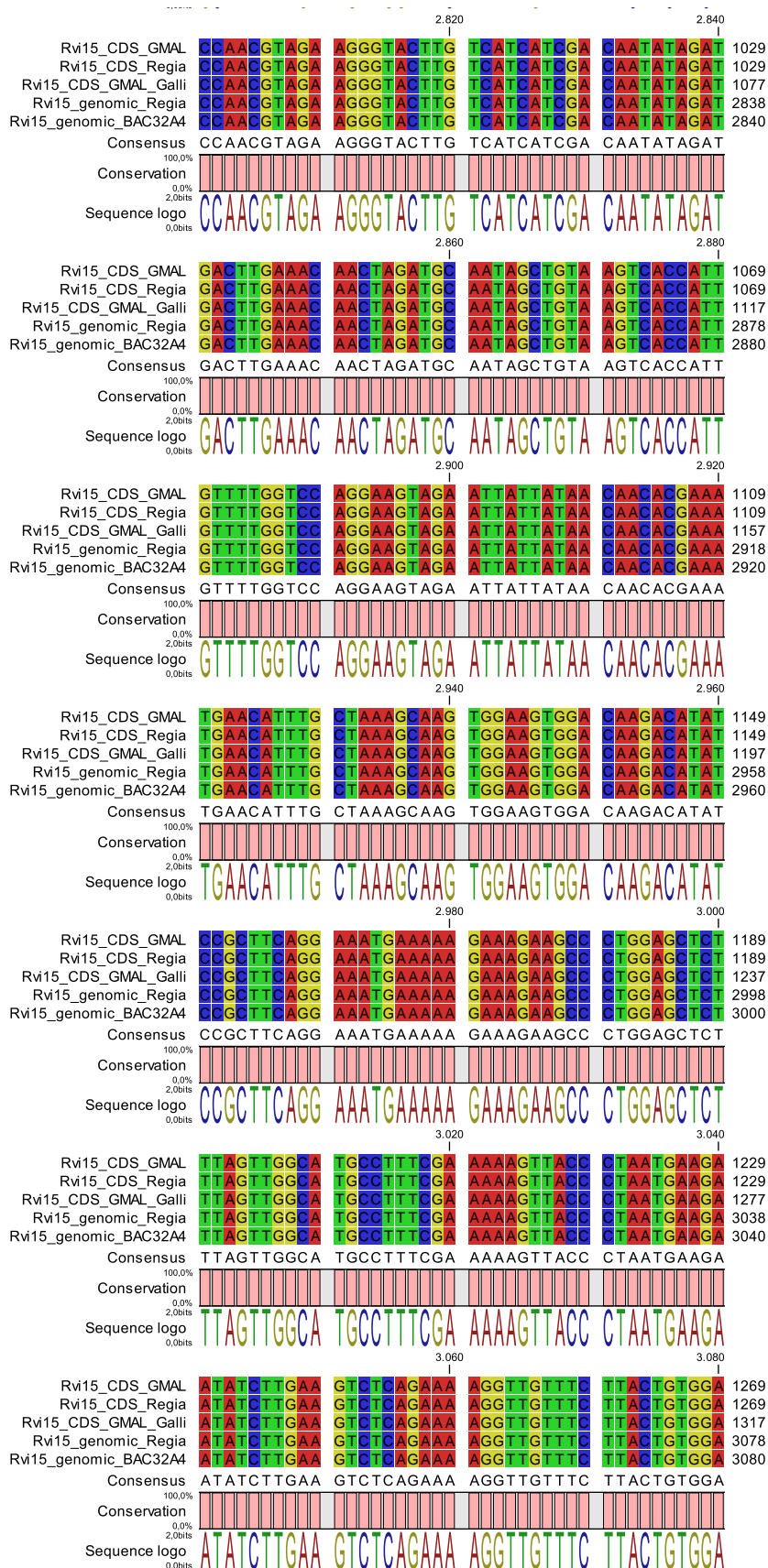

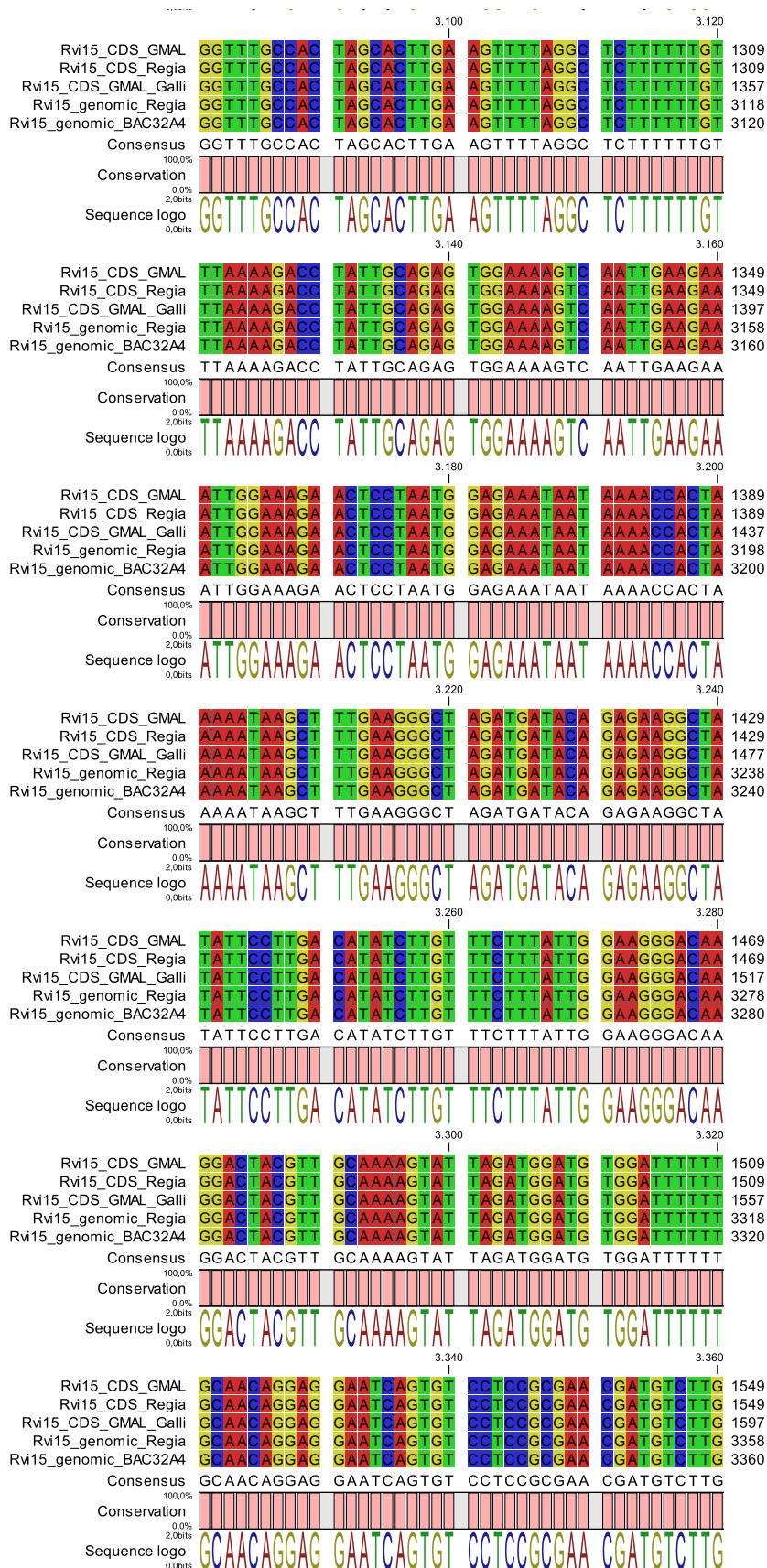

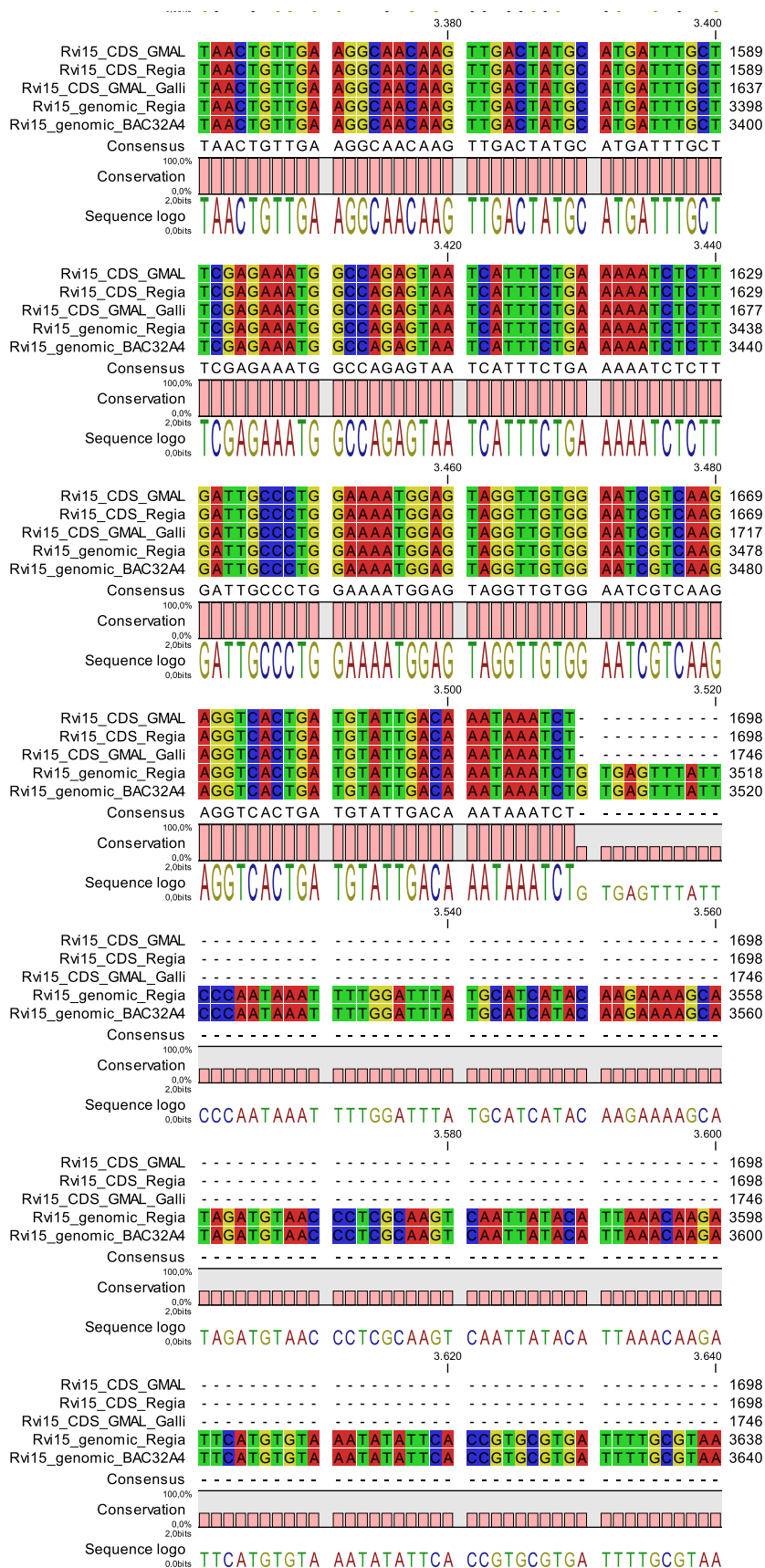

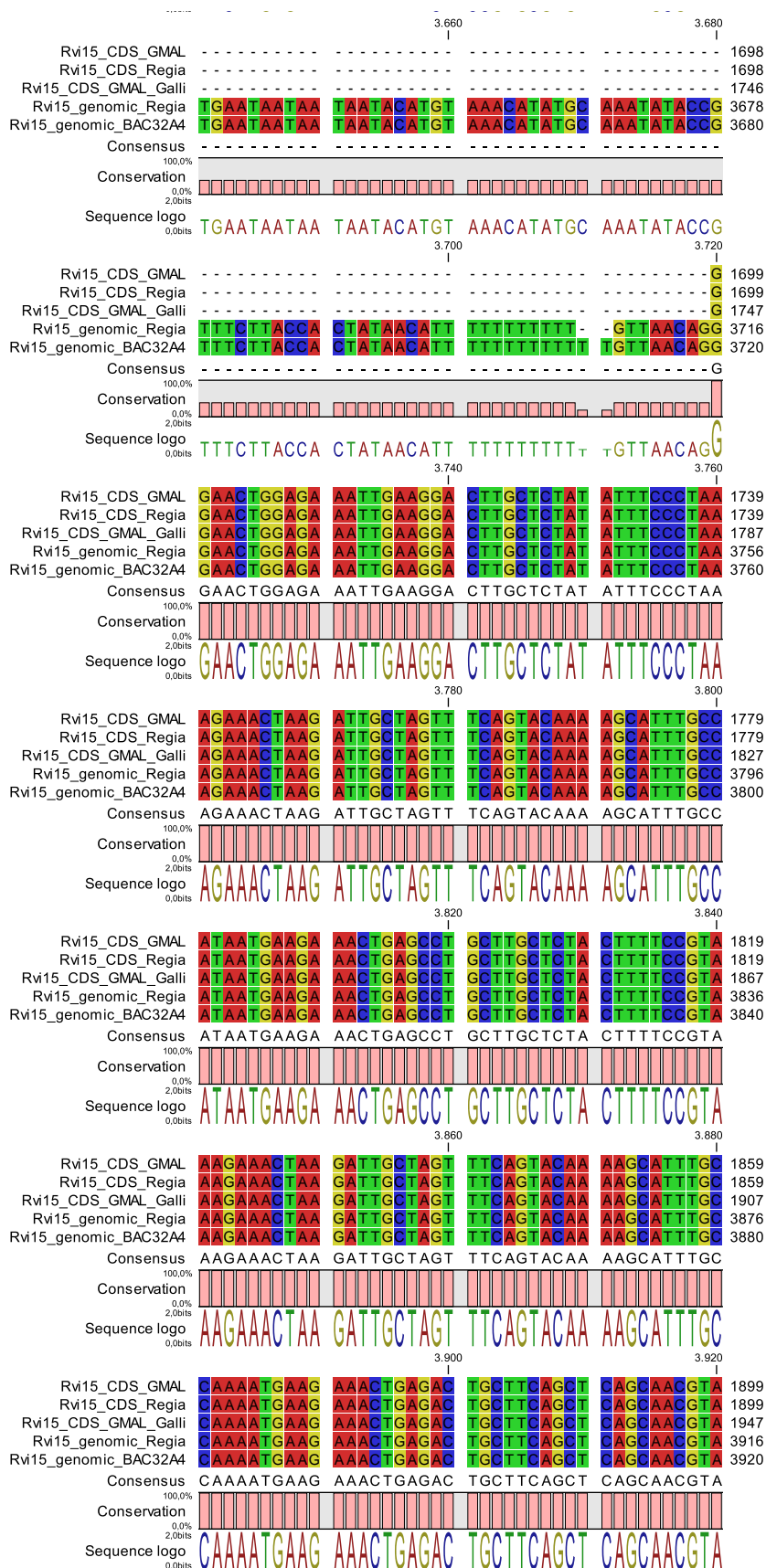

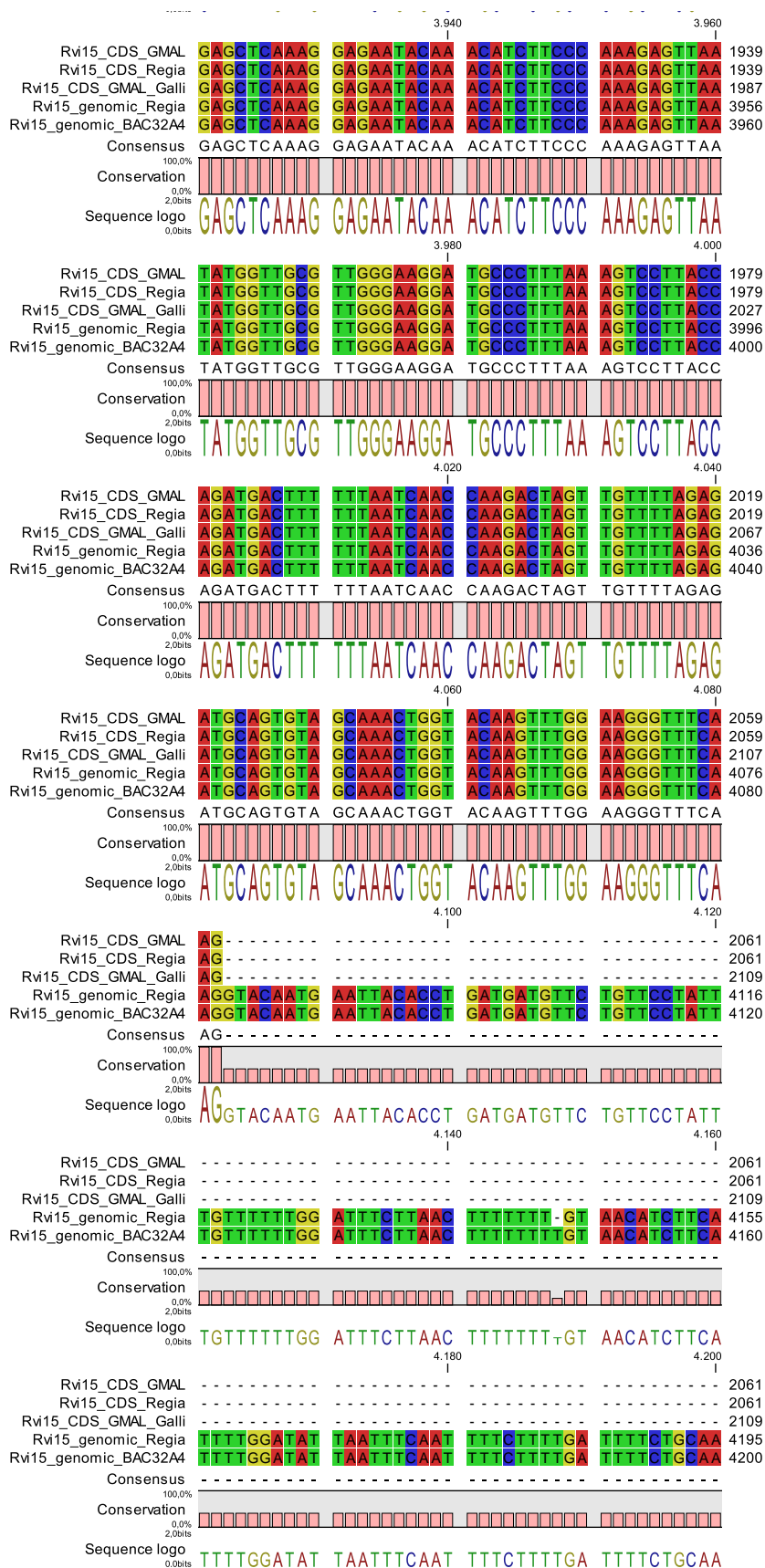

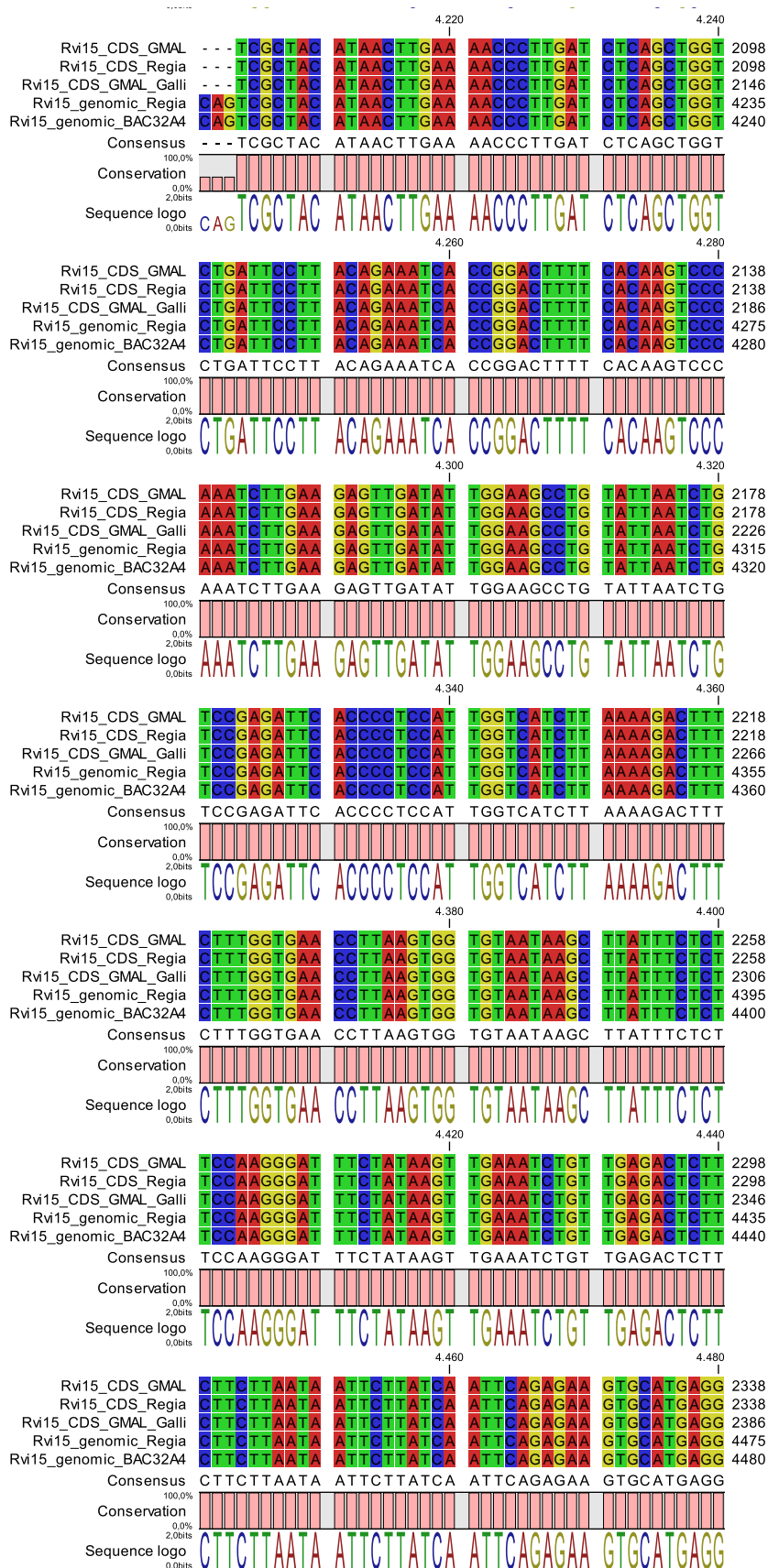

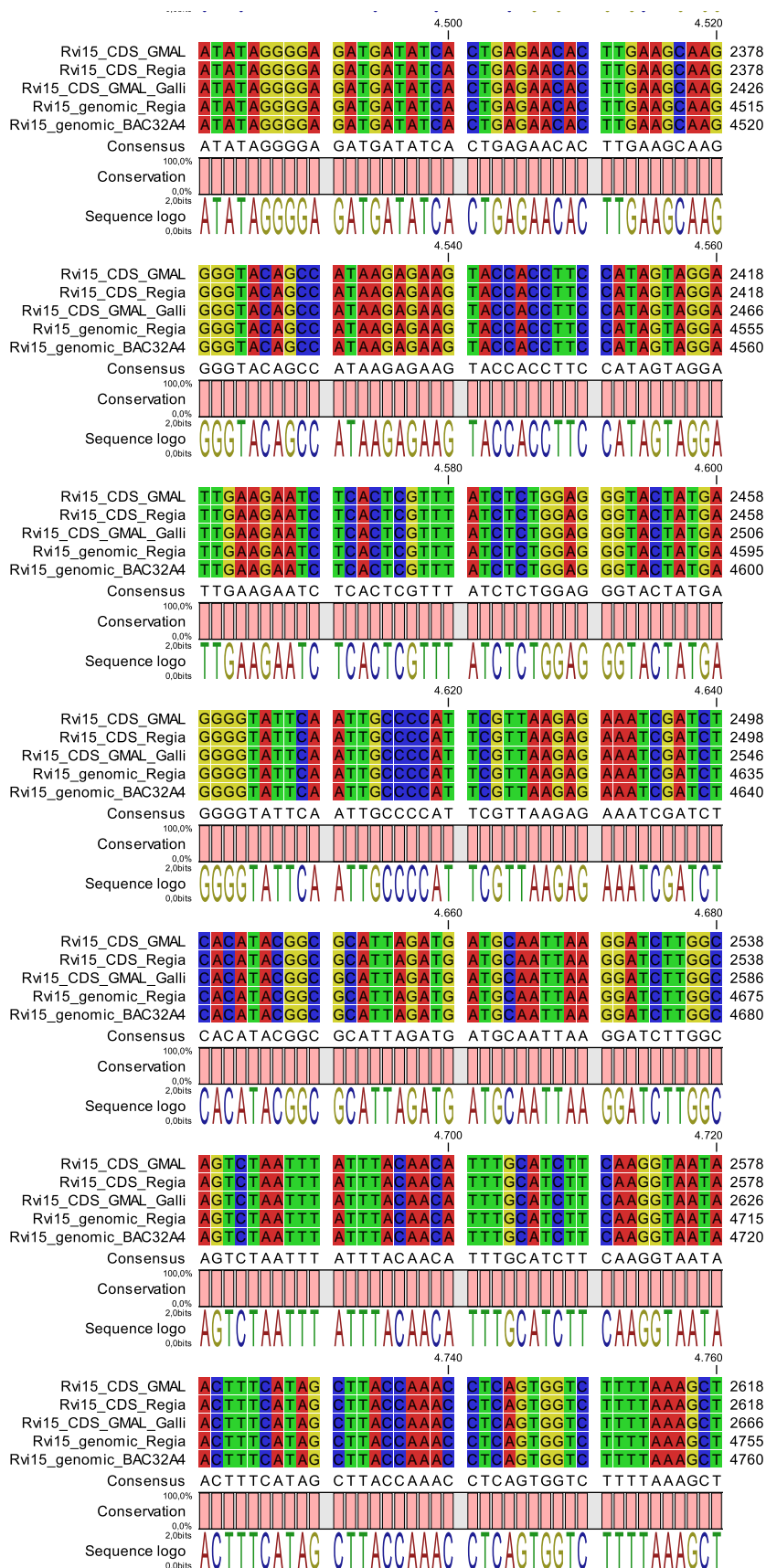

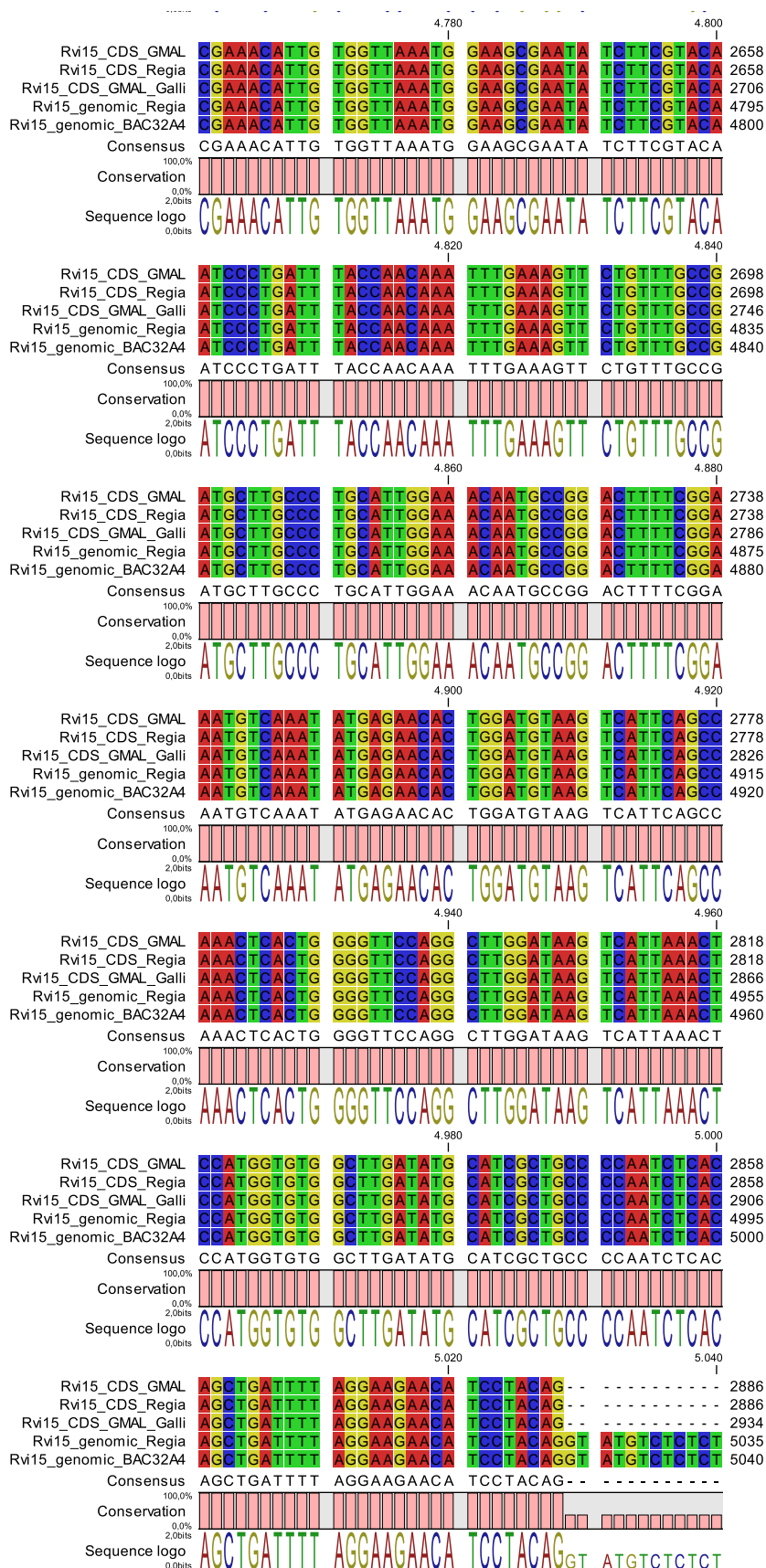

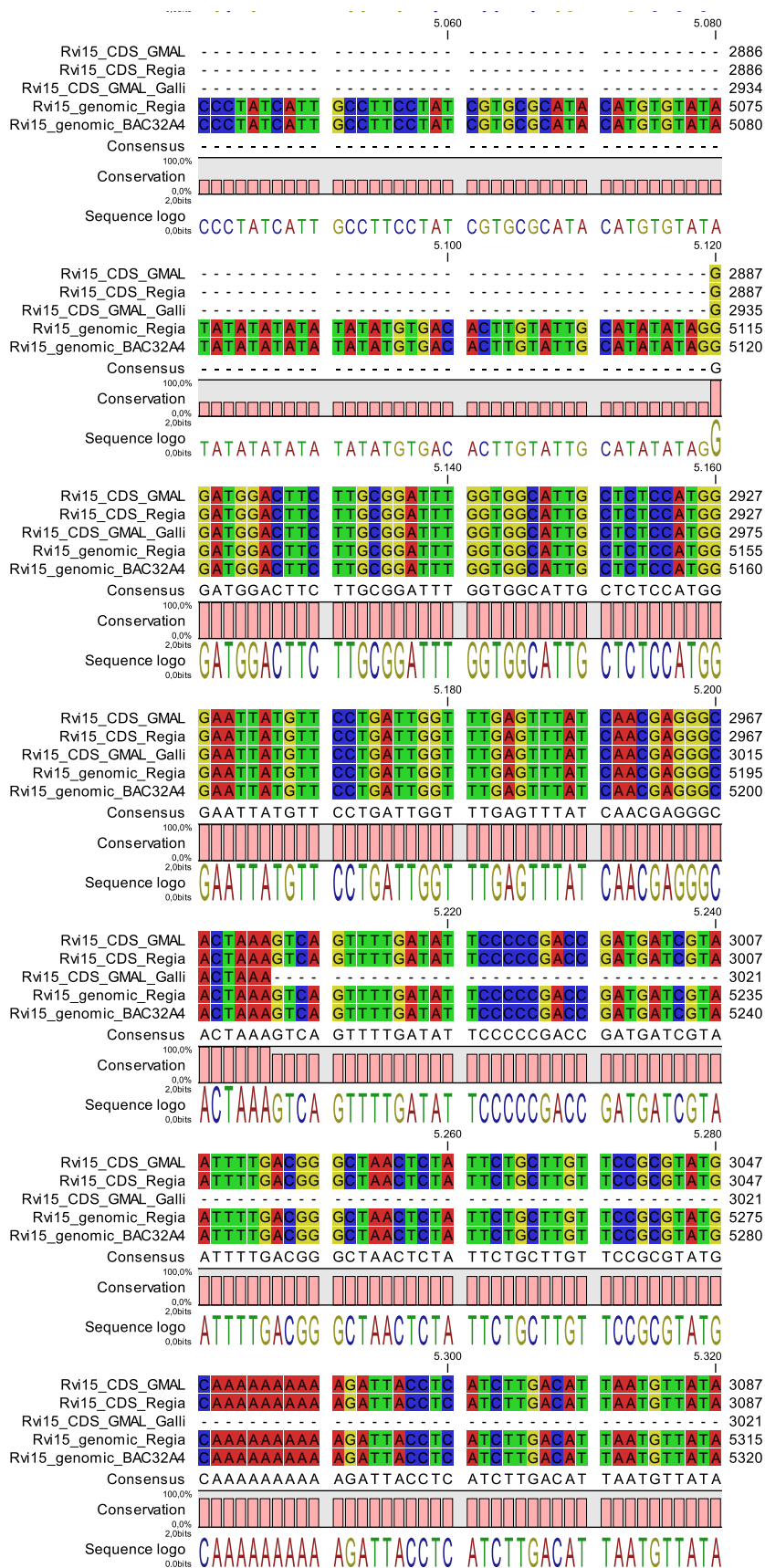

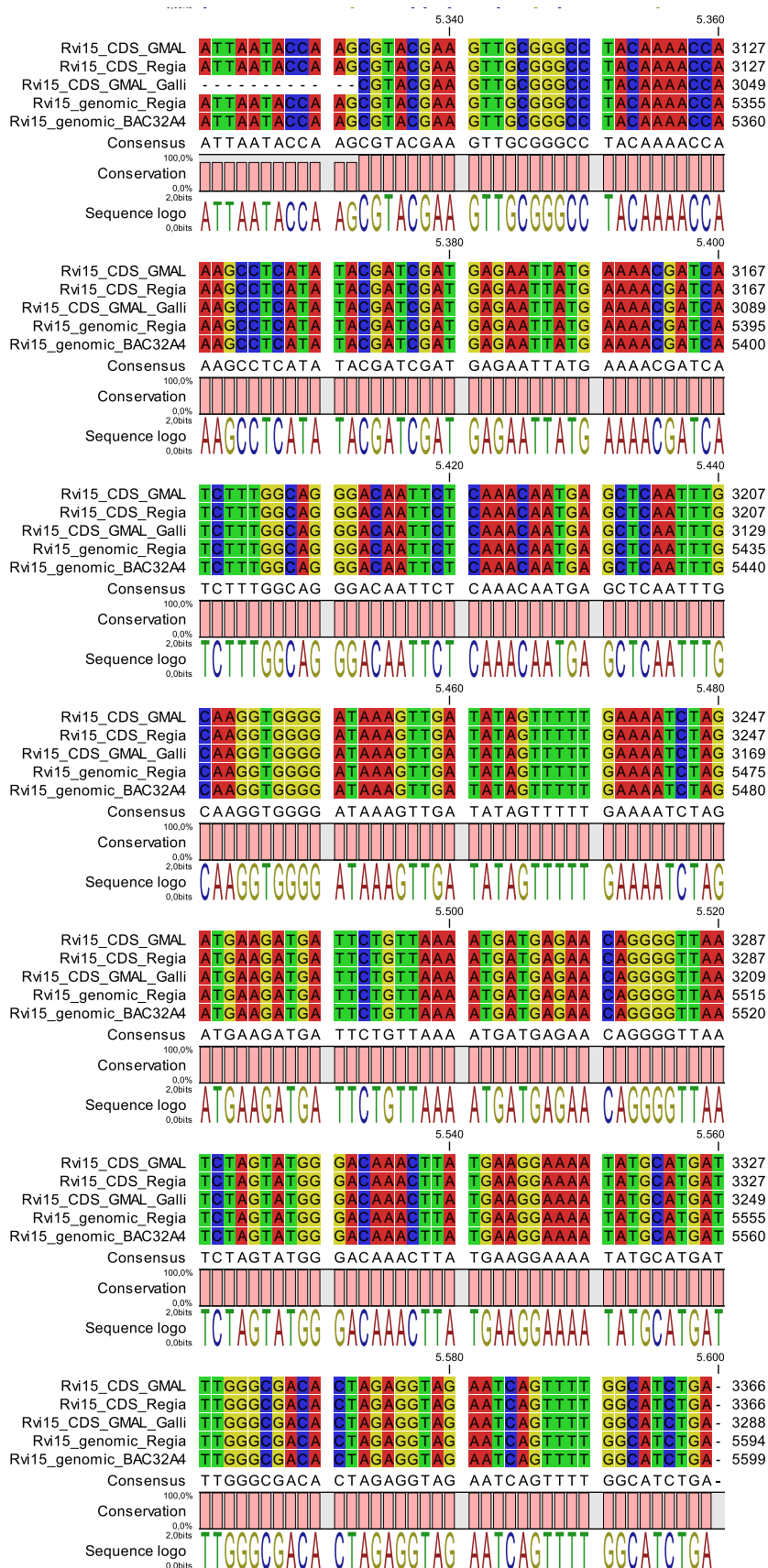

Supplement: Supplementary file 3 — Supplementary Table 6. Results of the artificial scab inoculation of the single replicates of the genotypes previously reported to carry Rvi4 or Rvi15 or a combination of both genes. 'Gala' was used as susceptible control. Online Resource 4. (PDF 9635 kb) [file 11032_2023_1421_MOESM3_ESM.pdf]
